# *Arabidopsis thaliana* EARLY RESPONSIVE TO DEHYDRATION 7 Localizes to Lipid Droplets via Its Senescence Domain

**DOI:** 10.3389/fpls.2021.658961

**Published:** 2021-04-14

**Authors:** Nathan M. Doner, Damien Seay, Marina Mehling, Siqi Sun, Satinder K. Gidda, Kerstin Schmitt, Gerhard H. Braus, Till Ischebeck, Kent D. Chapman, John M. Dyer, Robert T. Mullen

**Affiliations:** ^1^Department of Molecular and Cellular Biology, University of Guelph, Guelph, ON, Canada; ^2^United States Department of Agriculture, US Arid-Land Agricultural Research Center, Agriculture Research Service, Maricopa, AZ, United States; ^3^Department of Plant Biochemistry, Albrecht-von-Haller-Institute for Plant Sciences and Göttingen Center for Molecular Biosciences (GZMB), University of Göttingen, Göttingen, Germany; ^4^Department of Molecular Microbiology and Genetics, Institute for Microbiology and Genetics and Göttingen Center for Molecular Biosciences (GZMB), University of Göttingen, Göttingen, Germany; ^5^BioDiscovery Institute and Department of Biological Sciences, University of North Texas, Denton, TX, United States

**Keywords:** EARLY RESPONSIVE TO DEHYDRATION 7, lipid droplet, plant lipids, senescence domain, senescence domain-containing protein, senescence/spartin-associated domain, spartin, stress

## Abstract

Lipid droplets (LDs) are neutral-lipid-containing organelles found in all kingdoms of life and are coated with proteins that carry out a vast array of functions. Compared to mammals and yeast, relatively few LD proteins have been identified in plants, particularly those associated with LDs in vegetative (non-seed) cell types. Thus, to better understand the cellular roles of LDs in plants, a more comprehensive inventory and characterization of LD proteins is required. Here, we performed a proteomics analysis of LDs isolated from drought-stressed *Arabidopsis* leaves and identified EARLY RESPONSIVE TO DEHYDRATION 7 (ERD7) as a putative LD protein. mCherry-tagged ERD7 localized to both LDs and the cytosol when ectopically expressed in plant cells, and the protein’s C-terminal senescence domain (SD) was both necessary and sufficient for LD targeting. Phylogenetic analysis revealed that ERD7 belongs to a six-member family in *Arabidopsis* that, along with homologs in other plant species, is separated into two distinct subfamilies. Notably, the SDs of proteins from each subfamily conferred targeting to either LDs or mitochondria. Further, the SD from the ERD7 homolog in humans, spartin, localized to LDs in plant cells, similar to its localization in mammals; although, in mammalian cells, spartin also conditionally localizes to other subcellular compartments, including mitochondria. Disruption of *ERD7* gene expression in *Arabidopsis* revealed no obvious changes in LD numbers or morphology under normal growth conditions, although this does not preclude a role for ERD7 in stress-induced LD dynamics. Consistent with this possibility, a yeast two-hybrid screen using ERD7 as bait identified numerous proteins involved in stress responses, including some that have been identified in other LD proteomes. Collectively, these observations provide new insight to ERD7 and the SD-containing family of proteins in plants and suggest that ERD7 may be involved in functional aspects of plant stress response that also include localization to the LD surface.

## Introduction

Cells store neutral lipids, such as triacylglycerols (TAG) and sterol esters, in cytoplasmic lipid droplets (LDs), an organelle found in all kingdoms of life (Chapman et al., 2013; Lundquist et al., 2020). LDs are much more than simply lipid storage sites however, since they participate in a vast array of other cellular processes ranging from metabolism, protein turnover, signaling molecule biosynthesis, protein sequestration, and stress response (Pyc et al., 2017b; Kretzschmar et al., 2018; Olzmann and Carvalho, 2019; Lundquist et al., 2020). Underlying the control of these various processes are numerous LD “coat” proteins that reside on the surface of LDs and can be involved also in the formation of LDs at the endoplasmic reticulum (ER), mediating LD-LD or LD-organelle interactions, and LD turnover (Chapman et al., 2019; Thiam and Dugail, 2019; Ischebeck et al., 2020). While many LD proteins are conserved among evolutionarily-diverse organisms, some are unique to mammals, yeasts, or plants. Furthermore, the composition of LD coat proteins can vary even within the same species, depending on the tissue/cell type, developmental stage, and/or environmental cues. In *Arabidopsis thaliana*, for example, LD protein constituents differ in siliques, seeds, and seedlings (Kretzschmar et al., 2020), as well as in senescent (Brocard et al., 2017) or pathogen-challenged leaves (Shimada et al., 2014; Fernández-Santos et al., 2020).

Lipid droplets in plants are probably best known for their hyperaccumulation in oilseeds as a means to store carbon and energy. Once seed germination takes place, the stored lipids in oilseed LDs are catabolized to provide energy for seedling establishment until photoautotrophic growth can sustain the plant. However, in most other plant organs and tissues, including those that are typically not associated with lipid storage, such as in leaves, stems, and roots, the functions of LDs go beyond energy storage and include processes related to plant growth and development, lipid signaling, and biotic and abiotic stress responses (Pyc et al., 2017b; Ischebeck et al., 2020). Further, oleosins, which are involved with oilseed LD biology and are arguably the best characterized plant LD proteins, are not commonly found in vegetative tissues (Huang, 1996; Laibach et al., 2015). Our understanding of LD functions in non-seed tissues is limited, therefore, because relatively few LD-associated proteins have been studied in non-seed cell types. Nonetheless, recent efforts using mass-spectrometry (MS)-based proteomic analyses of purified LDs have led to the identification and subsequent characterization of several new plant-specific LD proteins. Pertinent examples include the LD-ASSOCIATED PROTEINS (LDAPs), which are required for proper LD biogenesis and neutral lipid homeostasis in vegetative tissues, including during stress responses (Gidda et al., 2013, 2016; Horn et al., 2013; Kim et al., 2016); PLANT UBX DOMAIN-CONTAINING PROTEIN 10 (PUX10), which is a member of the UBX-domain-containing protein family and is involved in LD protein degradation in *Arabidopsis* (Deruyffelaere et al., 2018; Kretzschmar et al., 2018); LDAP-INTERACTING PROTEIN (LDIP), which functions, presumably with LDAPs, in LD biogenesis (Pyc et al., 2017a; Coulon et al., 2020); and PHYTOALEXIN-DEFICIENT 3 (PAD3), which participates in the biosynthesis of antifungal compounds and localizes to leaf LDs during pathogen challenge (Fernández-Santos et al., 2020). As such, LD proteomics-based studies have not only provided important new insights into LD functioning in plants, they have led to the growing appreciation in the field that more LD proteins and the allied LD processes in which they are involved are yet to be discovered.

While LDs are known to proliferate during various biotic and abiotic stress treatments (reviewed in Xu and Shanklin, 2016; Pyc et al., 2017b; Yang and Benning, 2018; de Vries and Ischebeck, 2020; Lu et al., 2020), the linkage between LDs and stress responses is poorly understood, due in part to the limited numbers of LD-related proteins identified to date. Gene expression studies, on the other hand, have identified numerous genes that are differentially regulated during stress adaptation. Among these is EARLY RESPONSIVE TO DEHYDRATION 7 (ERD7), which is strongly upregulated by both biotic and abiotic stresses and, thus, commonly used as a marker for plant stress response (Cheng et al., 2013; Rasmussen et al., 2013). However, there is limited information on the function(s) of ERD7 and its subcellular localization in plant cells, with the exception of a recent study that demonstrated that ERD7 localizes in Arabidopsis protoplasts to membranes, albeit of uncertain identity (Barajas-Lopez et al., 2020). In the same study, ERD7 was shown to also bind to negatively-charged phospholipids *in vitro* and the composition of membrane lipids and membrane fluidity were altered in leaves of cold-stressed *erd7* mutant plants, suggesting that ERD7 plays a role in membrane lipid remodeling during cold stress response in Arabidopsis (Barajas-Lopez et al., 2020). ERD7 contains a predicted senescence domain (SD). The SD was originally named based on a protein that is upregulated in senescent daylily petals (Panavas et al., 1999), but is also found in other proteins in plants, metazoans, and fungi. Notably, in humans, the SD-containing protein spartin has been shown to localize to LDs (Eastman et al., 2009; Urbancyzk and Enz, 2011), as well as other subcellular compartments, including endosomes or mitochondria (Bakowska et al., 2007; Edwards et al., 2009; Joshi and Bakowska, 2011; Karlsson et al., 2014). While the spartin SD has been shown to bind the mitochondrial-associated phospholipid cardiolipin, which possibly serves as a mechanism for its mitochondrial localization (Joshi and Bakowska, 2011), how spartin targets to LDs and what its function(s) is on the LD surface are open questions.

In this study, the aim was to continue efforts to identify novel LD coat proteins in plant vegetative (non-seed) tissues. Toward that end, we analyzed the LD-enriched proteins isolated from drought-stressed Arabidopsis leaves, which is known to result in an accumulation of TAG and LD proliferation in plant vegetative tissues (Martin et al., 1986; Kong et al., 2013; Mukhopadhyay and Tyagi, 2015; Perlikowski et al., 2016; VanBuren et al., 2017; and reviewed in Xu and Shanklin, 2016; Yang and Benning, 2018; Lu et al., 2020). Among the various LD-related proteins we identified was ERD7, which we subsequently show using fluorescence microscopy localizes to LDs in plant cells. We also show that the protein’s SD mediates its LD localization. Interestingly, among the six SD-containing proteins in Arabidopsis, the SDs of ERD7 and both of its two closest homologs convey LD targeting in plant cells, as does the SD in human spartin, while the SD of other, more distantly-related Arabidopsis SD-containing proteins target to mitochondria. We discuss these and other findings, including a yeast two-hybrid (Y2H) screen that identified numerous stress-related proteins, and how they will provide new avenues for investigating the underlying mechanisms linking LDs and stress responses in plants.

## Materials and Methods

### Plant Material, Growth Conditions, and Transformations

Experiments performed with *A. thaliana* involved the wild-type (WT) Columbia-0 (Col-0) ecotype, an *erd7-1* T-DNA insertional mutant line obtained from the Arabidopsis Biological Resource Center [ABRC; WiscDsLox452E10 (seed stock CS856515)], an *erd7-2* CRISPR/Cas9-based deletion mutant line, and an ERD7-mCherry stable expression line (see ERD7-mCherry-Expressing and CRISPR/Cas9 erd7 Knockout Arabidopsis Lines section for additional details on the latter two transgenic plant lines). Soil-grown Arabidopsis plants were cultivated with a 16/8-h day/night cycle at 22°C and a light intensity of 50 μmol m^−2^ s^−1^. For growth on agar plates, seeds were sterilized with ethanol and plated on ½ strength Murashige and Skoog (Sigma-Aldrich) plates, and then stratified for 3 days at 4°C in the dark before being moved to a growth chamber with a 16/8-h day/night cycle at 22°C and a light intensity of 50 μmol m^−2^ s^−1^. Arabidopsis (stable) transformations were carried out using the floral dip method (Clough and Bent, 1998) and *Agrobacterium tumefaciens* (strain GV3101) harboring the selected binary vector (see Molecular Cloning and Plasmid Construction section for details on all binary vectors used in this study), as described elsewhere (Clough and Bent, 1998; Petrie et al., 2010).

*Nicotiana benthamiana* plants were grown in soil with a 16/8-h day/night cycle at 22°C and a light intensity of 50 μmol m^−2^ s^−1^. Leaves of ~4-week-old plants were transiently (co)transformed by infiltration with *A. tumefaciens* (strain LBA4404) carrying the selected binary vector(s), as described previously (McCartney et al., 2005; Cai et al., 2015). All (co)infiltrations with *N. benthamiana* were performed with the tomato bushy stunt virus (TBSV) *P19* gene in order to enhance ectopic gene expression (Petrie et al., 2010).

Suspension-cultured Arabidopsis cells were grown and transformed *via* biolistic bombardment, as described previously (Lingard and Trelease, 2006). Tungsten microparticles were coated with 10 μg of binary plasmid DNA [i.e., pMDC32-ChN/mCherry-ERD7 (see Molecular Cloning and Plasmid Construction section)] and 4 μg pRTL2/mGFP (Shockey et al., 2006), which encodes a monomerized version of the green fluorescent protein (GFP) and served as a cell (co)transformation marker. Approximately 24 h following bombardment, cells were prepared for confocal laser-scanning microscopy (CLSM), as described below (Microscopy section).

### ERD7-mCherry-Expressing and CRISPR/Cas9 *erd7* Knockout Arabidopsis Lines

To generate Arabidopsis Col-0 plants stably-expressing ERD7-mCherry, selected hygromycin-resistant T_1_ seedlings obtained after *Agrobacterium*-mediated plant transformation with pMDC32/ERD7-mCherry binary vector were advanced and resulting T_2_ progeny were analyzed for ERD7-mCherry fluorescence using CLSM. Unfortunately, for reasons unknown, we were unable to generate homozygous T_3_ lines stably-expressing ERD7-mCherry and, thus, experiments in this study were limited to assessing the subcellular localization of ERD7-mCherry in T_2_ lines.

Arabidopsis *erd7-2* mutant plants were generated using CRISPR/Cas9 genome editing of Col-0 based on the procedures described by Wang et al. (2015). Briefly, a pBEE401E-based vector containing the egg-cell-expressed *CAS9* open reading frame (ORF) and a pair of single-guide RNAs (sgRNAs) corresponding to sequences near the 5' and 3' ends of the *ERD7* ORF was introduced into Arabidopsis using the floral dip method (Clough and Bent, 1998); refer to Molecular Cloning and Plasmid Construction section for additional details on the construction of the pBEE401E-based vector used for CRISPR/Cas9 genome editing of *ERD7* and Supplementary Table 1 for sequences of sgRNAs and all other oligonucleotide primers used in this study. Genomic DNA (gDNA) of BASTA [i.e., phosphinothricin (Gold Biotechnology)]-resistant T_1_ plants was screened for the loss of the *ERD7* ORF using the PCR and gene-specific primers (Supplementary Table 1) and selected *erd7* mutant plants were advanced. gDNA from T_2_ progeny plants was then screened by PCR [and with the appropriate primers (Supplementary Table 1)] for the *CAS9*-containing T-DNA insert in order to identify plant lines that had lost the T-DNA through genetic segregation. Thereafter, edited *erd7* mutant and *CAS9*-deficient T_3_ plants were analyzed by PCR genotyping and deletion of a sequence encoding 398 amino acids of the 452-amino-acid-long ERD7 polypeptide was confirmed by sequencing of the *ERD7* PCR product. Refer to Supplementary Figure 1 for details on the relative locations of the sgRNAs and the region of the *ERD7* ORF that was removed by CRISPR/Cas9 genome editing in the *erd7-2* mutant line and the position of the T-DNA insertion in the *erd7-1* mutant line, as well as the genotyping and reverse transcriptase (RT)-PCR results for both mutant lines.

All DNA sequencing carried out in this study, including the sequencing of newly-constructed plasmids (see Molecular Cloning and Plasmid Construction section), was performed at the University of Guelph Genomics Facility or by Retrogen Inc.

### Proteomics of Drought-Treated Arabidopsis Leaves

Wild-type (Col-0) Arabidopsis were grown on soil for 4 weeks as described above before watering was withheld for 1 week. Three biological replicates of rosette leaves were harvested, and then LDs were isolated and the associated proteins subjected to liquid chromatography-tandem MS (LC-MS/MS), as described previously (Schmitt et al., 2017; Kretzschmar et al., 2020), but with the following modifications: centrifugation steps were carried out at 100,000 *g*, and the buffer used for grinding and all washing steps was supplemented with 0.4 M sucrose. Three technical replicates of LC-MS/MS analysis were performed on the peptide samples of each biological replicate.

The libraries, raw data files, MaxQuant search files, as well as protein groups and peptide search results created by MaxQuant are available on ProteomeXchange/PRIDE (Vizcaíno et al., 2014) under the identifier PXD023047. All LC-MS/MS data processing settings with MaxQuant can be found in the metadata file (Supplementary Table 2) and all raw and normalized/sorted proteomics data are shown in Supplementary Datasets 1 and 2, respectively.

### Molecular Cloning and Plasmid Construction

The *ERD7* ORF was amplified from Arabidopsis rosette leaf cDNA using PCR and primers [i.e., ERD7-FP-*Sal*I and ERD7-RP-*Xho*I (Supplementary Table 1)] and then, following digestion with *Sal*I and *Xho*I, subcloned into similarly-digested donor vector pENTR3C (Invitrogen), yielding pENTR3C/ERD7. The *ERD7* ORF was subsequently transferred from pENTR3C/ERD7 into pMDC32-ChN using Gateway cloning technology (Curtis and Grossniklaus, 2003), yielding pMDC32/Ch-ERD7. pMDC32-ChN is a plant expression binary vector which contains two copies of the constitutive *35S* Cauliflower mosaic virus (CaMV) promoter, the ORF of the red fluorescent protein mCherry, and an adjacent 3' cloning site that allows for the translational (in-frame) fusion of mCherry to the N-terminus of a protein of interest (i.e., mCherry-X). To construct pMDC32-ChN, the mCherry ORF (without its stop codon; Shaner et al., 2004) was amplified by PCR from pRTL2/mCherry (Gidda et al., 2011) using primers mCherry-FP-*Kpn*I and mCherry-RP-*Asc*I (Supplementary Table 1) and inserted (*via Kpn*I/and *Asc*I restriction sites) upstream of the recombination site in pMDC32 (Curtis and Grossniklaus, 2003). To construct pMDC32/ERD7-mCherry, encoding ERD7 fused at its C-terminus to mCherry, primers ERD7-FP-pDONR and ERD7-RP-pDONR(NS) (Supplementary Table 1) were used to amplify the *ERD7* ORF from Arabidopsis rosette leaf cDNA. Thereafter, the *ERD7* ORF was transferred to pDONR-zeo and then pMDC32-ChC (Kretzschmar et al., 2020), *via* Gateway cloning.

All ERD7 mutants were amplified using pENTR3C/ERD7 (see above) as template DNA and the appropriate primers (Supplementary Table 1). Resulting PCR products were transferred into pMDC32-ChN using Gateway cloning, sometimes without being subcloned into the intermediate pDONR vector, as described in Müller et al. (2017) and referred to here as Fast Gateway cloning. *SENA2*, *SENA3*, *SENB1*, and *SENB2* full-length ORFs and their corresponding SD sequences were amplified from Arabidopsis rosette leaf cDNA using the appropriate gene-specific primers (Supplementary Table 1), followed by Gateway or Fast Gateway cloning into pMDC32-ChC. The coding region containing the SD in human (*Homo sapiens*) spartin [i.e., spartin-SD; amino acid residues 418–623, based on the InterPro domain database (see Bioinformatics section; accession no. NM_015087.5)] was amplified from cDNA derived from cultured human dopaminergic neurons using primers SPARTINSD-FP-pDONR and SPARTINSD-RP-pDONR (Supplementary Table 1) and cloned into pMDC32-ChC *via* Fast Gateway cloning. A full-length human spartin cDNA (CloneID HsCD00743790) was obtained from the DNASU Plasmid Repository^1^ and used as a template, along with the appropriate primers, for Fast Gateway cloning into pMDC32-ChC. Note that compared to the reference sequence for spartin (i.e., NM_015087.5), the full-length spartin clone used in this study contained three point mutations (accession no. LT742893.1), resulting in two amino acid changes, P69T and P417H, both of which are outside of the protein’s SD.

The construction of pMDC43/GFP-LPEAT1, encoding GFP-tagged Arabidopsis ACYL CoA:LYSOPHOSPHATIDYLETHANOLAMINE ACYLTRANSFERASE isoform 1 (LPEAT1), and pORE04/P19, encoding the Tomato bushy student virus (TBSV) RNA-silencing suppressor P19, has been described elsewhere (Petrie et al., 2010; Jasieniecka-Gazarkiewicz et al., 2021). The binary plasmid encoding the GFP-tagged mitochondrial marker protein (i.e., GFP-mito) was obtained from ABRC (plasmid stock CD3-987) and consists of the N-terminal mitochondrial targeting peptide from *Saccharomyces cerevisiae* cytochrome c oxidase subunit 4 fused to GFP (Nelson et al., 2007).

For Y2H screening with ERD7 (see Yeast-Two-Hybrid section), the Arabidopsis ERD7 ORF was amplified by PCR using appropriate primers (Supplementary Table 1), and then PCR products were digested with *Eco*RI and *Bam*HI, and subcloned into similarly-digested pGBKT7-DNA-BD (Clontech), yielding the “bait” vector pGBKT7/ERD7.

The pBEE401E-based vector used for CRISPR/Cas9 genome editing of Arabidopsis *ERD7* was constructed based on procedures described by Wang et al. (2015). Briefly, sgRNAs were designed using CRISPR-PLANT v2^2^ (Minkenberg et al., 2019) to specifically target the *ERD7* ORF (refer to Supplementary Figure 1 for the relative positions of the sgRNA regions targeted for CRISPR/Cas9 genome editing of the *Arabidopsis ERD7* gene). Primers ERD7-sgRNA1 and ERD7-sgRNA2 (Supplementary Table 1) were synthesized to contain each sgRNA sequence, as well as sequences complementary to the pCBC-DT1T2 template plasmid. A region of pCBC-DT1T2 (containing the sgRNA scaffold, promoters, and terminators) was then amplified and the resulting PCR products were digested with *Bsa*I, gel purified, and ligated using Golden Gate cloning into similarly-digested pBEE401E (Wang et al., 2015), yielding the binary vector pBEE401E/ERD7sgRNA.

### Immunoblotting

Immunodetection of mCherry-tagged ERD7 protein in *Agrobacterium*-infiltrated *N. benthamiana* leaves 3 days post-infiltration was carried out as follows. Leaf material was snap-frozen in liquid nitrogen and ground into powder in extraction buffer consisting of 100 mM Tris-HCl pH 7.5, 150 mM NaCl, 10% (v/v) glycerol, 0.1% (v/v) Tween-20, 1 mM PMSF, and Protease Inhibitor Cocktail (Sigma-Aldrich). Extracts were clarified by centrifugation and the soluble (total) protein fraction was incubated with SDS Sample buffer (Laemmli, 1970) and boiled. Thereafter, proteins were separated by SDS-PAGE and electroblotted onto Hybond® nitrocellulose (GE LifeSciences). Membranes were stained with 0.5% (w/v) Ponceau S (Sigma-Aldrich) in 1% acetic acid, visualized, and then destained with ddH_2_0, blocked (and subsequently washed) in TBST buffer [20 mM Tris-HCl pH 7.5, 150 mM NaCl, 0.05% (v/v) Tween-20] containing 3% (w/v) skim milk powder, followed by incubations with anti-red fluorescent protein (RFP) antibodies, which recognize the mCherry protein (Invitrogen), and anti-mouse IgG-peroxidase antibodies (Sigma-Aldrich). Immunoreactive proteins were visualized using Amersham ECL Prime Western Blotting Detection Reagent, according to the manufacturer’s instructions (GE Healthcare), and a ChemiDoc MP (Bio-Rad Laboratories).

### RT-PCR and Genotyping

*ERD7* gene expression in different WT Arabidopsis organs and tissues was assessed by RT-PCR. Plant material was snap-frozen and ground to a powder. RNA was then extracted using TRIzol reagent (Invitrogen) and an EZ-10 spin column plant RNA kit (Bio Basic Inc.), followed by cDNA synthesis using 750 ng RNA and qScript cDNA SuperMix (Quantabio). One microliter of cDNA was used per 20 μl reaction which consisted of 30 cycles of 30 s at 94°C, 45 s at 55°C, and 90 s at 72°C. *ERD7* and Arabidopsis β-TUBULIN isoform 4 (*TUB4*) serving as a reference gene, were amplified using gene-specific primers (Supplementary Table 1).

Genotyping and RT-PCR analysis of the Arabidopsis *erd7-1* and *erd7-2* mutants were carried out as follows. gDNA was isolated by grinding frozen rosette leaves from 4-week-old plants for each mutant line in extraction buffer [200 mM Tris-HCl pH 7.5, 250 mM NaCl, 25 mM EDTA, 0.5% (w/v) SDS] and then, following centrifugation, the supernatant was mixed 1:1 with isopropanol. Thereafter, samples were subjected to centrifugation, the resulting pellet washed with 70% ethanol and resuspended in 10 mM Tris-HCl pH 7.5, and PCR was performed using the appropriate gene-specific primers (Supplementary Table 1). RT-PCR to confirm the absence of *ERD7* transcript in the *erd7-1* and *erd7-2* mutant lines was performed as described above.

### Microscopy

Wild-type and transgenic Arabidopsis seeds and leaves, *Agrobacterium*-infiltrated and drought-stressed *N. benthamiana* leaves, and biolistically-bombarded Arabidopsis suspension-cultured cells were processed for CLSM imaging, including staining of LDs with neutral lipid-selective fluorescent dyes boron-dipyrromethene (BODIPY) 493/503 (Invitrogen; Listenberger et al., 2007) and monodansylpentane (MDH; Abcepta; Yang et al., 2012), as previously described (Cai et al., 2015; Gidda et al., 2016).

Imaging of BODIPY-stained LDs in leaves of drought-stressed Arabidopsis plants, as described in Proteomics of Drought-Treated Arabidopsis Leaves section, was performed with a Zeiss LSM780 CLSM (Carl Zeiss Microscopy GmbH). All other imaging performed in this study was carried out using a Leica SP5 CLSM equipped with a Radius 405-nm laser (Leica Microsystems). Excitations and emission signals for fluorescent (mCherry or GFP) fusion proteins, BODIPY, and/or MDH were collected sequentially as single optical sections or *Z*-series in double-labeling experiments as described previously (Gidda et al., 2016). Single-labeling experiments showed no detectable crossover at the settings used for data collection. All fluorescence images of plant cells shown in individual figures are representative of at least three separate experiments, including >30 transformed *N. benthamiana* leaf cells and >10 Arabidopsis suspension cells; except for images of leaf cells from drought-stressed (and control) Arabidopsis plants whereby 10 leaf areas (i.e., two leaf areas from five different plants) per treatment were analyzed. Numbers and/or diameters of LDs in Arabidopsis seedling, senescing leaves, and drought-stressed (and control) leaves were quantified according to Cai et al. (2015), using the Analyze Particles function in ImageJ (v1.8.0; Schneider et al., 2012), and statistically analyzed using Student’s *t*-test. All figure compositions were generated using Microsoft PowerPoint (Microsoft).

### Yeast-Two-Hybrid

Screening of a Y2H library, consisting of Arabidopsis cDNA from various plant tissues and cloned into the appropriate prey vector, using Arabidopsis ERD7 [pGBKT7/ERD7 (see Molecular Cloning and Plasmid Construction section)] as “bait,” was carried out with the Matchmaker Gold Y2H System (Clontech Laboratories, Inc.), as described by the manufacturer and as we have done so in previously-published Y2H screens (Park et al., 2013; Pyc et al., 2017a). All yeast strains that grew on selective media [synthetic dextrose media lacking tryptophan and leucine but containing X-α-Gal and Aureobasidin A (Takara Bio United States Inc.)], were designated as either “strong,” “intermediate,” or “weak” interactors based on the relative color of the colony, which corresponds to the activation of the *MEL1* reporter gene. Plasmids were extracted from yeast cells to determine the identity of encoded candidate ERD7-interacting (prey) proteins (listed in Supplementary Table 3) by automated DNA sequencing.

### Bioinformatics

Annotations for the subcellular localizations of all detected proteins identified in proteomics analysis of drought-stressed Arabidopsis leaves were obtained from the Plant Proteome Database (PPDB^3^; Sun et al., 2009). The protein-protein network analysis of selected candidate LD-enriched proteins was performed using the Search Tool for Retrieval of Interacting Genes/Proteins (STRING) database^4^ (Szklarczyk et al., 2019), with default settings and a minimum required interaction score = 0.400. Protein sequences of SD-containing proteins, including ERD7, in Arabidopsis and other plant species were obtained from The Arabidopsis Information Resource (TAIR^5^; Berardini et al., 2004), UniProt,^6^ or the Phytozome 12 database.^7^ The SD in each protein was identified and the amino acid sequence defined based on information provided at the InterPro domain database^8^ (Blum et al., 2021); note that the SD is referred to as senescence/spartin-associated domain. Sequences of ERD7 homologs in non-plant species (and their corresponding SDs), including human spartin, were obtained also from the InterPro database. Phylogenetic analyses were carried out using MEGA-X software (Kumar et al., 2018) and the phylogenetic tree was constructed using the neighbor-joining method. Microarray data for the relative expression of *ERD7* and selected *SEN* genes in Arabidopsis was obtained using the Arabidopsis electronic fluorescent pictographic (eFP) Browser hosted at The Bio-Analytic Resource for Plant Biology (BAR^9^; Kilian et al., 2007; Winter et al., 2007) and corresponding heat maps were generated using Microsoft Excel (Microsoft). Multiple sequence alignments used to calculate amino acid sequence identities/similarities between proteins were generated using Clustal Omega^10^ (Madeira et al., 2019). Gene Ontology (GO) Slim terms were identified using the GO annotation search at TAIR. Putative amphipathic helices were predicted using the HeliQuest server^11^ (Gautier et al., 2008), mitochondrial targeting sequences were predicted using the DeepLoc^12^ (Almagro Armenteros et al., 2017) and Plant-mPLoc^13^ (Chou and Shen, 2010) servers, and lipidation was predicted using the GPS-Lipid server^14^ (Xie et al., 2016).

## Results

### ERD7 Is Enriched With LDs Isolated From Drought-Stressed Arabidopsis Leaves

As an extension of our recent efforts to identify and characterize new LD proteins in plant vegetative (i.e., non-seed) tissues (Horn et al., 2013; Cai et al., 2015; Gidda et al., 2016; Pyc et al., 2017a; Kretzschmar et al., 2018, 2020; Greer et al., 2020), mature Arabidopsis plants were subject to 1 week of drought treatment in order to elicit TAG accumulation and LD proliferation of LDs, as reported previously in drought-stressed plants (reviewed in Xu and Shanklin, 2016; Yang and Benning, 2018; Lu et al., 2020). Consistent with this, we observed a significant increase in the number of LDs stained with the neutral lipid-selective dye BODIPY 493/503 in leaves from drought stress-treated vs. control (i.e., watered) Arabidopsis plants (Supplementary Figure 2). LDs were isolated (in triplicate) from leaves of stress-induced plants and proteins from LD-enriched fractions, as well as those from the corresponding total cellular extracts, were analyzed using quantitative MS based on the label-free quantification (LFQ) algorithm (Cox and Mann, 2008; Cox et al., 2014), similar to our other previously-published Arabidopsis LD proteomics studies (Pyc et al., 2017a; Kretzschmar et al., 2018, 2020). All detected proteins are listed, both as the raw and normalized data, in Supplementary Datasets 1 and 2, respectively, with the latter dataset also showing each protein’s LFQ abundance and LD enrichment compared to the total cellular extracts (i.e., LD enrichment was calculated for each protein as the ratio of LFQ abundance in isolated LD fractions vs. total cellular extracts). Shown in Supplementary Dataset 3 are the summed LFQ and enrichment values for all detected proteins at different subcellular localizations based on PPDB (Sun et al., 2009; Ischebeck et al., 2020), including proteins annotated to be localized at LDs, which were the most enriched in the LD fractions, as expected. Among the other notable groups of proteins enriched in the LD fractions were those annotated to be localized to tonoplasts or vacuoles (Supplementary Dataset 3), which might reflect the LD-vacuole interactions that are proposed to occur during autophagic degradation of LDs in plants (Ischebeck et al., 2020; Zienkiewicz and Zienkiewicz, 2020), as well as proteins localized to lipid-storing plastoglobuli, which, like cytosolic LDs, are known to be upregulated during plant stress (Rottet et al., 2015; Lundquist et al., 2020).

We further categorized all of the detected proteins in drought-stressed Arabidopsis leaves [based on the normalized dataset (Supplementary Dataset 2)] to include only those that (i) were found in all each of the three (replicate) LD-enriched fractions, (ii) had a relative LFQ abundance >0.2, and (iii) had an LD-enrichment score of >10. The resulting cohort of 89 proteins that co-purified with LDs are shown in Supplementary Dataset 4 and a STRING-based protein-protein interaction network based on annotated functional associations and/or physical interactions of these proteins is shown in Supplementary Figure 3. Overall, the STRING network had three prominent clusters of proteins, including one enriched in vacuolar ATPase subunits, which was consistent with the enrichment of proteins in the LD fractions annotated (at PPBD) to be localized to vacuoles or tonoplasts (Supplementary Table 3). The two other main clusters of proteins in the network were enriched in translation-related proteins (e.g., ribosomal subunits and eukaryotic initiation factors) or stress-related proteins, the latter also including several well-known LD proteins, i.e., proteins experimentally confirmed *via* microscopy to be localized to LDs in plant cells (based on Ischebeck et al., 2020). To illustrate this further, all of the known LD proteins and the top candidate LD-associated proteins (based on Supplementary Dataset 4) are shown in Table 1. Notably, several of the known LD proteins that were either significantly enriched or only detected in LD-enriched fractions from drought-stressed Arabidopsis leaves (Table 1) have been also previously associated with plant stress, such as CALEOSIN 3 [CLO3, also referred to as RESPONSIVE TO DESSICATION 20 (RD20; Aubert et al., 2010; Park et al., 2018)], LDAP1 and LDAP3 (Gidda et al., 2016; Kim et al., 2016), and α-dioxygenase 1 (DOX1; Shimada et al., 2014). Also among the proteins exclusively detected in LD-enriched fractions were several previously found in other Arabidopsis LD proteomics studies but have not been experimentally characterized in terms their association with LDs and/or LD-related function(s). Examples of these proteins are shown also in Table 1 and included several of the abovementioned vacuolar ATPase subunits that were also found in the LD proteomes in senescent (Brocard et al., 2017) and/or pathogen (i.e., *Pseudomonas*)-challenged leaves (Fernández-Santos et al., 2020). In addition, several stress-related proteins were exclusively detected in LD-enriched fractions, including the dehydrin family members ERD10 and COLD-REGULATED 47, as well as ERD7 (Table 1), which, as discussed in the Introduction section, was selected for further study for several reasons.

**Table 1:** Known and candidate lipid droplet (LD) proteins enriched or only detected in LD fractions isolated from drought-stressed *Arabidopsis* leaves.

Name	AGI No.	Description	Total rLFQ	LD rLFQ	Other LD proteomes
Known LD proteins					
CLO3	AT2G33380	Caleosin 3/responsive to desiccation 20	0.606	37.96	Ref. 1
LDAP1	AT1G67360	Lipid droplet-associated protein 1	-	2.577	“
LDAP3	AT3G05500	Lipid droplet-associated protein 3	-	0.747	“
DOX1	AT3G01420	α-Dioxygenase 1	-	0.698	“
CAS1	AT2G07050	Cycloartenol synthase 1	-	0.209	“
LDIP	AT5G16550	LDAP-interacting protein	-	0.112	“
LIME	AT4G33110	Putative methyltransferase	-	0.085	“
Other proteins only found in LD fractions					
VHA-H	AT3G42050	Vacuolar ATP synthase subunit H family protein	-	2.305	Ref. 3–5
VHA-A3	AT4G39080	Vacuolar proton ATPase A3	-	1.647	Ref. 2, 3
VHA-D1	AT3G28710	ATPase, V_0_/A_0_ complex, subunit C/D	-	1.503	Ref. 3, 4
ERD10	AT1G20450	Dehydrin family protein	-	1.106	Ref. 3
COR47	AT1G20440	Cold-regulated 47	-	1.091	Ref. 3
NCL	AT1G53210	Sodium/calcium exchanger family protein	-	0.956	Ref. 2, 3, 5
EF1B	AT5G19510	Translation elongation factor EF1β	-	0.728	Ref. 4, 5
VAB2	AT4G38510	ATPase, V1 complex, subunit B protein	-	0.633	Ref. 3–5
AT3G57020	AT3G57020	Calcium-dependent phosphotriesterase superfamily protein	-	0.611	Ref. 4–6
MC4	AT1G79340	Metacaspase 4	-	0.575	Ref. 3, 4
AT4G17390	AT4G17390	Ribosomal protein L23/L15e family protein	-	0.573	Ref. 4, 5
VAM3	AT5G46860	Syntaxin/t-SNARE family protein	-	0.566	Ref. 3
CYP89A9	AT3G03470	Cytochrome P450, family 87, subfamily A, polypeptide 9	-	0.520	Ref. 3, 4
SS2	AT1G74020	Strictosidine synthase 2	-	0.518	Ref. 3, 5
AT3G17020	AT3G17020	Adenine nucleotide α-hydrolases-like superfamily	-	0.508	Ref. 3–5
VMA10	AT3G01390	Vacuolar membrane ATPase complex, subunit G	-	0.478	Ref. 3, 5
CHC1	AT3G11130	Clathrin heavy chain 1	-	0.464	Ref. 3, 4
ERD7	AT2G17840	Senescence/dehydration-associated protein-related	-	0.459	Ref. 2, 3
AVP1	AT1G15690	Inorganic H pyrophosphatase family protein	-	0.452	Ref. 3, 5
PATL3	AT1G72160	Sec14p-like phosphatidylinositol transfer family protein	-	0.449	Ref. 3
SYTA	AT2G20990	Synaptotagmin A	-	0.421	Ref. 2, 3, 5

All detected proteins identified in drought-stressed *Arabidopsis* leaves in this study are listed in Supplementary Datasets 1 and 2, along with their respective LFQ abundance and LD enrichment compared to the total cellular extracts. All of the known and candidate LD-associated proteins that were enriched or only detected in LD fractions are also listed in Supplementary Dataset 4. Shown for each protein is its name (symbol), Arabidopsis Genome Initiative (AGI) identifier number, and description, as assigned by TAIR. Shown in the upper portion of the table are known (i.e., experimentally confirmed *via* microscopy) LD proteins, based on Ischebeck et al. (2020) and indicated as (Ref. 1), that were either significantly enriched (i.e., CLO3) or only detected (e.g., LDAP1/3) in LD-enriched fractions based on Supplementary Dataset 2. Shown in the lower portion of the table are the most abundant proteins in Supplementary Dataset 4 found exclusively in LD-enriched fractions and which were also found in other previously-published *Arabidopsis* LD proteomics studies, including pathogen-challenged leaves (Fernández-Santos et al., 2020; Ref. 2), senescing leaves (Brocard et al., 2017; Ref. 3), and/or seeds or seedlings (Kretzschmar et al., 2020; Ref. 4; Kretzschmar et al., 2018; Ref. 5; Pyc et al., 2017a; Ref. 6). Relative label-free quantification (rLFQ) values reflect the protein abundance in the LD-enriched fractions (LD rLFQ) and the corresponding total cellular extracts (Total rLFQ) as measured by the LFQ algorithm (Cox and Mann, 2008; Cox et al., 2014), and normalized so that the abundance of all detected proteins sum to 1000 (i.e., rLFQ). Dashes in the Total rLFQ column indicate proteins that were not detected in total cellular extracts.

### Ectopically-Expressed ERD7 Is Localized to LDs and Cytosol in Plant Cells

**Figure 1:**
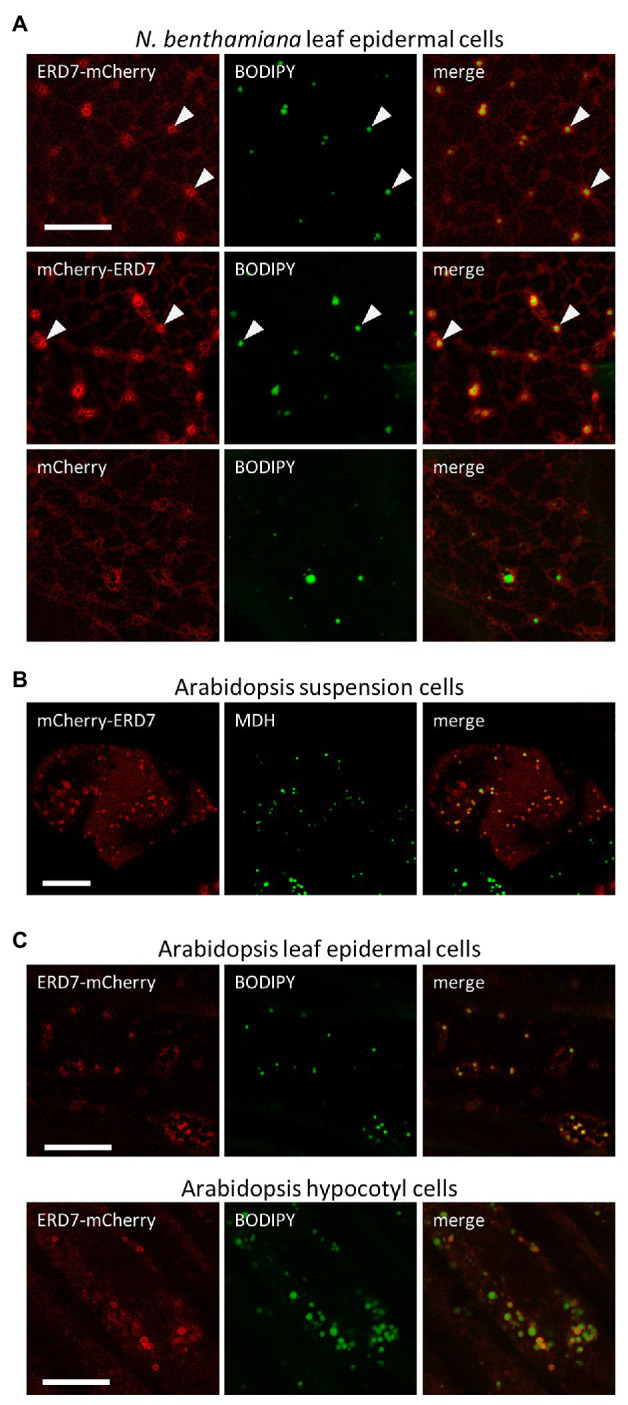
Subcellular localization of EARLY RESPONSIVE TO DEHYDRATION 7 (ERD7) in plant cells. Representative confocal laser-scanning microscopy (CLSM) images (z-sections) of either **(A)** *Agrobacterium*-infiltrated *Nicotiana benthamiana* leaf epidermal cells, **(B)** biolistically-bombarded *Arabidopsis* suspension-cultured cells, or **(C)** transgenic *Arabidopsis* seedling leaf epidermal and hypocotyl cells, transiently- or stably-transformed with ERD7-mCherry, mCherry-ERD7, or mCherry alone (as indicated by the panel labels), and stained with the neutral lipid dyes BODIPY or MDH (false-colored green). Also shown for each set of images is the corresponding merged image. Arrowheads in the top two rows in **(A)** indicate obvious examples of mCherry-ERD7 and ERD7-mCherry fluorescence patterns surrounding the BODIPY-stained TAG core, indicating that ERD7 localizes to the surface of LDs. Scale bars in **(A–C)** = 10 μm.

To further evaluate the subcellular localization of ERD7 in plants, the protein was translationally appended, either at its N- or C-terminus, to the fluorescent protein mCherry (i.e., mCherry-ERD7 and ERD7-mCherry) and then transiently expressed in *N. benthamiana* leaves, which is a well-characterized model system for studying plant protein subcellular localization (Sparkes et al., 2006; Petrie et al., 2010). As shown in Figure 1A, CLSM imaging revealed that both of the ERD7 fusion proteins displayed similar fluorescence patterns in transformed *N. benthamiana* leaf (epidermal) cells, including distinct torus-shaped fluorescence patterns that encircled the BODIPY-stained LDs. While this staining pattern is reminiscent of other LD-related proteins transiently expressed in plant cells (Gidda et al., 2016; Pyc et al., 2017a; Kretzschmar et al., 2020), a portion of both ERD7 fusion proteins also displayed a more diffuse-like fluorescence pattern, suggesting localization to the cytosol. Indeed, a similar diffuse fluorescence pattern was observed in *N. benthamiana* leaf cells transiently transformed with mCherry alone (Figure 1A). Furthermore, there was no obvious localization of ERD7 to the ER in *N. benthamiana* leaf cells, i.e., mCherry-ERD7 did not readily colocalize with a co-expressed GFP-LPEAT1 serving as an ER marker protein (Jasieniecka-Gazarkiewicz et al., 2021; Supplementary Figure 4A). We also performed immunoblotting with anti-mCherry antibodies to confirm that both mCherry-ERD7 and ERD7-mCherry transiently expressed in *N. benthamiana* leaves were of the expected molecular mass (i.e., ~77 kDa; Supplementary Figure 4B), indicating that the cytosolic localization observed for both fusion proteins was not simply due to cleavage of the appended mCherry moiety.

We also assessed the subcellular localization of mCherry-tagged ERD7 in Arabidopsis, the native (plant) environment for the ERD7 protein. As shown in Figure 1B, mCherry-ERD7 localized predominantly to MDH-stained LDs in transiently-transformed suspension-cultured Arabidopsis cells, although a portion of the fusion protein was also apparent in the cytosol, as in *N. benthamiana* leaf cells (Figure 1A). Similarly, ERD7-mCherry localized to BODIPY-stained LDs and the cytosol in both leaf epidermal and hypocotyl cells in stably-transformed Arabidopsis seedlings (Figure 1C). Collectively, these data support the results from our proteomics studies (Table 1) that ERD7 is localized to LDs, but further suggest that the expression levels of ERD7 and/or other factors might influence its distribution between LDs and the cytosol.

### The LD Targeting Information in ERD7 Is Located in Its SD

**Figure 2:**
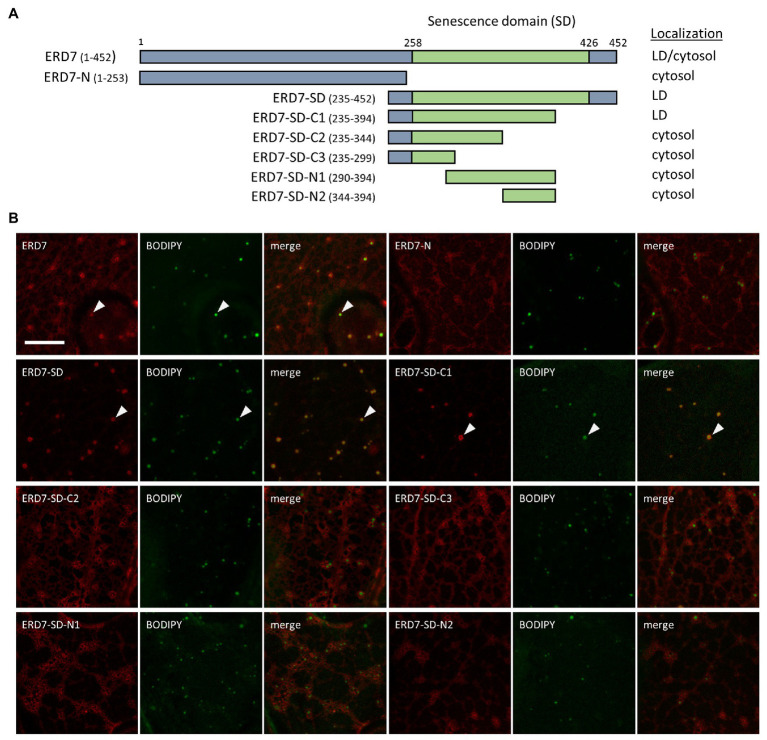
Subcellular localization of various ERD7 truncation mutants in *N. benthamiana* leaves. *N. benthamiana* leaves were transiently transformed (*via Agrobacterium* infiltration) with either full-length or truncated versions of ERD7 and tagged at their N-termini with mCherry. Three days post-infiltration, leaves were stained with the neutral lipid dye BODIPY and imaged using CLSM. Shown in **(A)** are schematic representations of full-length ERD7 and the various ERD7 mutants and their corresponding subcellular localization(s) [i.e., cytosol and/or lipid droplet (LD)]. Numbers above the illustration of full-length ERD7 represent specific amino acids residues, and the SD (amino acid residues 258–426) is defined based on information obtained from the InterPro database. The numbers next to the name of each construct denote the amino acid residues in ERD7 that were fused to mCherry; note that the N-terminal-appended mCherry moiety is not depicted in the illustrations or construct names. The portion of ERD7 representing the SD or the other parts of the protein are depicted in the illustrations as green or gray boxes, respectively. Shown in **(B)** are representative CLSM images (z-sections) of each mCherry-ERD7 (mutant) protein, as indicated by the panel labels, along with the corresponding BODIPY-stained LDs in the same cell. Also shown is the corresponding merged image. Arrowheads indicate examples of mCherry-ERD7 and the selected (mutant) versions thereof (i.e., ERD7-SD and ERD7-C1) that localized to BODIPY-stained LDs; compare with the lack of LD localization for the other mutant versions of ERD7. Scale bar in **(B)** = 10 μm.

In order to identify the putative LD targeting information in ERD7, we carried out a mutational analysis of the protein. As depicted in Figure 2A, we initially generated mCherry-tagged ERD7 mutants consisting of either the N-terminal [amino acids 1–253 (referred to as ERD7-N)] or C-terminal (amino acids 235–452) halves of ERD7, the latter of which being referred to as ERD7-SD since it included the protein’s SD (amino acids 258–426). Each fusion construct was then transiently expressed in *N. benthamiana* leaves and their localization to BODIPY-stained LDs (or a lack thereof) was assessed using CLSM. As shown in Figure 2B, ERD7-N localized to the cytosol, with no apparent targeting to BODIPY-stained LDs. By contrast, ERD7-SD localized almost exclusively to LDs (Figure 2B), i.e., all of the fluorescence attributable to ERD7-SD in *N. benthamiana* leaf cells appeared to be enriched at BODIPY-stained LDs, with relatively little fluorescence observed in the cytosol (compare with images of ERD7-N and full-length ERD7 in Figures 1A, 2B). As shown also in Figure 2, additional fusion constructs consisting of different regions of the C-terminal half of ERD7 all mislocalized to the cytosol, with the exception of ERD7-C1, which consisted of amino acids 235–394, including the majority of the SD (fused to mCherry), and, similar to ERD7-SD, localized exclusively to LDs. These data indicate that the LD targeting information in ERD7 is located within a relatively long C-terminal region of the protein, including most of the SD.

### SEN Proteins Are Phylogenetically Organized Into Two Distinct Subfamilies in Plants, Including in Arabidopsis

**Figure 3:**
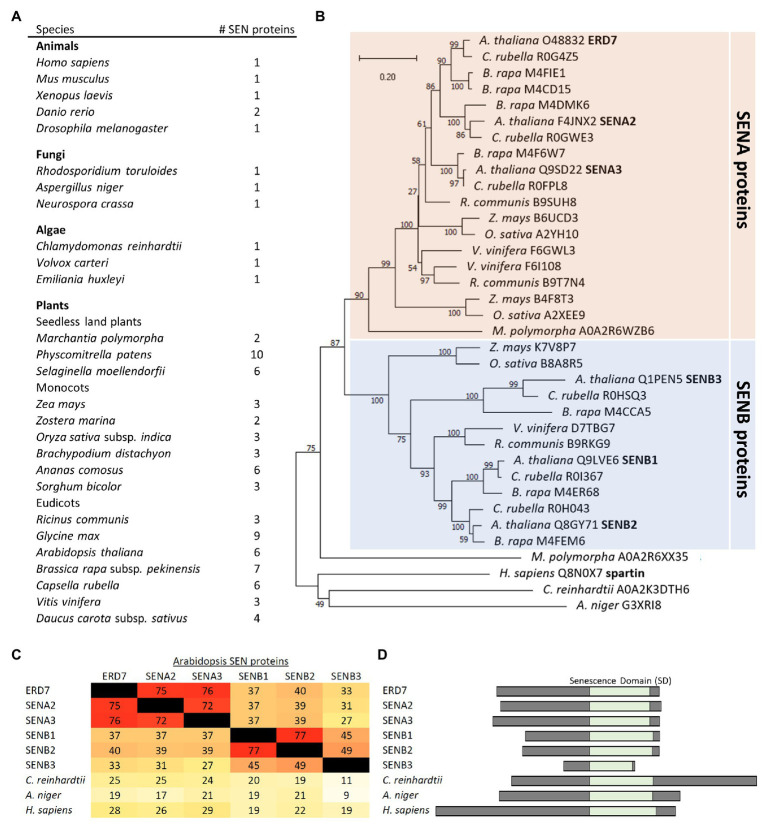
Comparison of SEN protein families in plants and other evolutionarily-diverse species. **(A)** Shown are the number of proteins in selected species from plants, animals, fungi, and algae that are annotated to contain an SD, based on the InterPro domain database for SDs (InterPro entry: IPR009686), and ruling out redundant entries and splice isoforms based on the UniProt annotations for each protein. **(B)** Phylogenetic tree based on the SD sequences of SEN proteins from selected plant and non-plant species, obtained from the Uniprot or Phytozome databases, and constructed with the neighbor-joining method using MEGA-X (Kumar et al., 2018). Bootstrap values are indicated beside each branch point. The scale bar represents the number of amino acid substitutions per site. Note that, with exception of one protein from *M. polymorpha*, all of the plant SEN proteins shown group into either the SENA or SENB protein subfamilies. Each protein is listed by its species and Uniprot accession number. Also indicated in bold are the six SENA/B proteins, including ERD7, in Arabidopsis, as well as human spartin. **(C)** A heat map displaying the primary sequence identity (as a percentage) among the SDs of the six Arabidopsis SENA/B proteins and selected SEN proteins from other non-plant species, all which were calculated based on a ClustalO multiple sequence alignment. Darker (red) colors reflect higher sequence identities, while lighter (yellow) colors reflect lower sequence identities, consistent with the corresponding percent identities shown in each panel. **(D)** Schematic representations of the six Arabidopsis SENA/B proteins, as well as selected SEN proteins from algae, fungi, and animals (i.e., *Chlamydomonas reinhardtii*, *Aspergillus niger*, and human), depicting the relative location (and length) of their SDs and adjacent N- and C-terminal regions.

Based on searches of the InterPro domain database, the SD is annotated to be of unknown function and found in proteins from a vast array of evolutionarily-diverse species, ranging from metazoans (animals), fungi, algae, and plants. Further, while most animal, fungal, and algal species have just one protein with an SD, multiple SD-containing proteins exist in land plants (Figure 3A), which we refer to collectively in all species as SENESCENCE DOMAIN-ASSOCIATED (SEN) proteins. For example, the SEN protein families in the seedless plant species *Marchantia polymorpha* and *Physcomitrella patens* possess two and 10 members, respectively. Monocots, such as *Zea mays* and *Oryza sativa*, each have three members, and Arabidopsis, a eudicot, has six members, including ERD7 (Figure 3A). These observations suggest that the expansion of the SEN protein families in land plants occurred early in their evolution and that they may have acquired additional novel attributes (e.g., functions, subcellular localization, etc.) compared to their SEN homologs in other (non-land plant) species.

Phylogenetic analysis, based on sequences of plant homologs found in the Phytozome 12 database or non-plant homologs from the UniProt database, further revealed that the plant SEN proteins generally form two distinct subfamilies, termed here SENA and SENB (Figure 3B). In Arabidopsis, the six SEN proteins are divided equally between each subfamily, with ERD7 (i.e., SENA1), SENA2, and SENA3 found in the SENA subfamily and SENB1-3 found in the SENB subfamily (Figure 3B). As depicted in Figure 3C, the C-terminal SDs in each of the Arabidopsis SENA and SENB proteins share their highest deduced amino acid sequence identities with the two other members of the same subfamily, while all of the Arabidopsis SENA/B proteins possess relatively low sequence identities with the SD sequences in their SEN protein homologs from non-plant species, such as *Chlamydomonas reinhardtii*, *Aspergillus niger*, and *H. sapiens*. The N-termini of the Arabidopsis SENB proteins are also generally shorter in length than those of their SENA protein counterparts, as well as those in SEN protein homologs from non-plant species (Figure 3D), with SENB3 having the shortest N-terminal sequence, as well as a C-terminal-truncated SD (Figure 3D).

### The SDs of the Arabidopsis SENA and SENB Proteins Target Specifically to LDs and Mitochondria, Respectively

**Figure 4:**
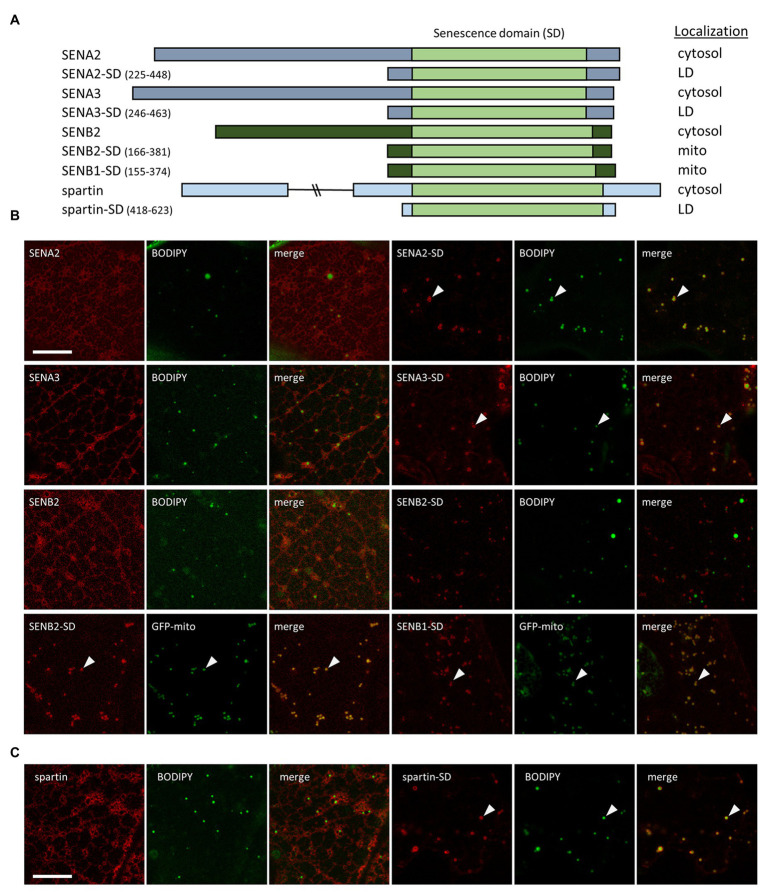
Subcellular localization of various Arabidopsis SEN proteins and human spartin, and their corresponding SDs. *N. benthamiana* leaves were transiently transformed (*via Agrobacterium* infiltration) with either full-length or the C-terminal portion, including their SD, of Arabidopsis SENA2, SENA3, SENB1, SENB2, or human spartin, and tagged at their C-termini with mCherry. Three days post-infiltration, leaves were stained with the neutral lipid dye BODIPY and imaged using CLSM. Alternatively, leaves were co-transformed with either SENB2-SD-mCherry or SENB1-SD-mCherry and the mitochondrial marker protein GFP-mito. **(A)** Schematic representations of the full-length and/or C-terminal SD-containing portion of each *Arabidopsis* SENA/B protein and human spartin, and their corresponding subcellular localization(s) [i.e., cytosol, lipid droplet (LD), mitochondria (mito)]. The SD in each protein is based on information obtained from the InterPro domain database. The numbers next to the name of each SD construct denote the amino acid residues that were fused to mCherry. The portion of each protein representing the SD is depicted in the illustrations as a light green box, while other regions of the SENA, SENB, and spartin proteins are depicted as gray, dark green, and blue boxes, respectively. Note that the C-terminal-appended mCherry moiety is not depicted in the illustrations or construct names. Representative CLSM images (z-sections) of mCherry-tagged full-length or truncated SEN-family proteins **(B)** or spartin **(C)**, along with the corresponding BODIPY-stained LDs or co-expressed GFP-mito in the same cell, and as indicated by panel labels. Also shown is the corresponding merged image. Arrowheads indicate examples of SENA/B or spartin fusion proteins that localized to BODIPY-stained LDs or GFP-mito-labeled mitochondria. Scale bars in **(B,C)** = 10 μm.

With the exception of ERD7, none of the other Arabidopsis SENA or SENB proteins were identified in our or other previously-published Arabidopsis LD proteomics studies (Brocard et al., 2017; Pyc et al., 2017a; Kretzschmar et al., 2018, 2020; Fernández-Santos et al., 2020) and all of these proteins, like ERD7, are annotated (based on TAIR) to have unknown function and subcellular localization. To determine the subcellular location of the other Arabidopsis SEN proteins and whether there might be differences between (or among) the members of each subfamily, full-length proteins or just the corresponding C-terminal portions, containing the SD regions, were appended to mCherry and transiently-expressed in *N. benthamiana* leaves. The subcellular localizations of the fusion proteins were then assessed using CLSM. As shown in Figures 4A,B, both full-length SENA2 and SENA3 localized to the cytosol, but not to BODIPY-stained LDs, unlike the LD and cytosolic localization observed for full-length ERD7 (Figures 1, 2). However, similar to the subcellular localization observed for ERD7-SD (Figure 2), the SENA2 and SENA3 C-terminal portions alone (i.e., SENA2-SD and SENA3-SD), which contain the SD, localized specifically to LDs (Figures 4A,B). Full-length SENB2 also localized only to the cytosol, but unlike the LD localization observed for the SD regions from the SENA proteins, SENB2-SD localized to punctate structures that were distinct from BODIPY-stained LDs (Figures 4A,B). Based on their size and shape, these structures were hypothesized to be mitochondria and, indeed, co-expression of SENB2-SD with a GFP-mitochondrial marker fusion protein (i.e., GFP-mito; Nelson et al., 2007) revealed that both proteins colocalized at mitochondria in *N. benthamiana* leaf cells (Figure 4B). Similarly, SENB1-SD colocalized with GFP-mito (Figure 4B), indicating that the SDs of the Arabidopsis SENB proteins contain mitochondrial targeting information.

We also assessed the subcellular localization of the human SEN protein, spartin, in *N. benthamiana* leaf cells in order to determine if this protein, similar to ERD7, might localize to LDs in plant cells, as it does in animal cells (Eastman et al., 2009; Urbancyzk and Enz, 2011). As shown in Figure 4C, transiently-expressed full-length spartin (fused to mCherry) localized to the cytosol, with no apparent localization to BODIPY-stained LDs nor to any other punctate structures [such as mitochondrial-localized SENB1-SD and SENB2-SD (Figure 4B)]. On the other hand, the region of spartin containing its SD alone fused to mCherry localized readily to LDs (Figure 4C), indicating that the LD targeting information in the SD region is likely conserved between Arabidopsis ERD7, SENA2, SENA3, and their human SEN protein counterpart, spartin. Further, in both sets of proteins, the LD targeting information is somewhat cryptic in nature, being more readily exposed when the N-terminal portion of each protein is removed.

### LDs in *erd7* Mutant and WT Arabidopsis Leaves and Seeds Are Similar in Their Numbers and Sizes Under Normal Growth Conditions

Prior characterizations of plant LD-associated proteins, particularly those involved with LD biogenesis or turnover, have revealed conspicuous alterations in LD abundance and/or morphology in cells when their expression in disrupted (Siloto et al., 2006; Gidda et al., 2016; Pyc et al., 2017a; Kretzschmar et al., 2018). Considering that ERD7 localizes to LDs in leaves (Figure 1), we investigated if disruption of *ERD7* expression might alter LD homeostasis under normal growth conditions. Toward that end, an Arabidopsis mutant, which contains a T-DNA insertion in *ERD7* and termed here *erd7-1*, was obtained from ABRC, genotyped and advanced to homozygosity, then confirmed (*via* RT-PCR) to be devoid of any full-length *ERD7* transcripts (Supplementary Figure 1). We also generated a second independent homozygous Arabidopsis *ERD7* knockout mutant (i.e., *erd7-2*) using CRISPR/Cas9 genome editing (see ERD7-mCherry-Expressing and CRISPR/Cas9 erd7 Knockout Arabidopsis Lines section in the Materials and Methods for details), which was employed to remove most of the *ERD7* coding sequence, resulting also in no detectable *ERD7* expression (Supplementary Figure 1).

**Figure 5:**
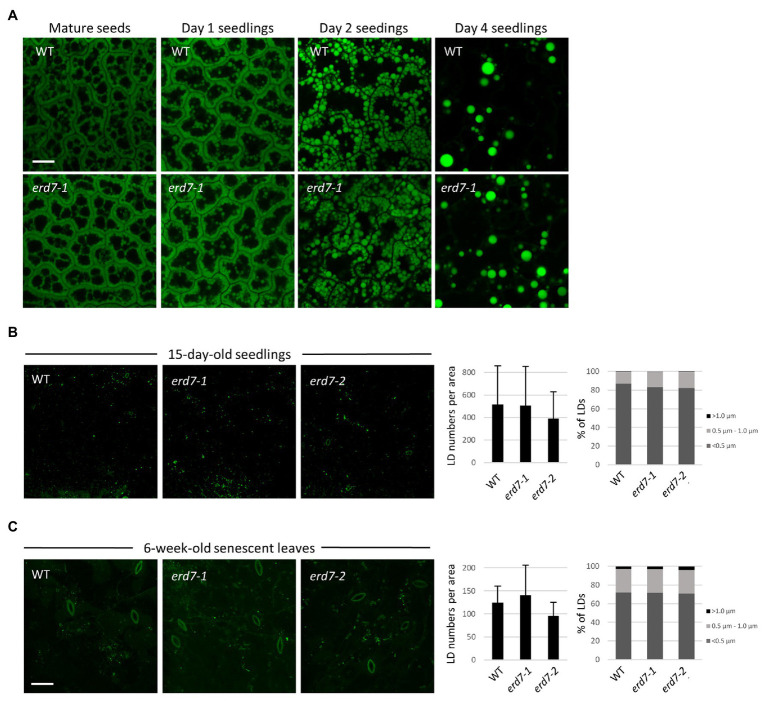
ERD7 does not appear to be required for regulating LD numbers and sizes in *Arabidopsis* under normal growth conditions. Shown are representative CLSM images of BODIPY-stained LDs in leaf epidermal cells of **(A)** embryo cotyledon cells in mature (dry) seeds and germinating seedlings either 1, 2, or 4 days after germination; **(B)** 15-day-old *Arabidopsis* seedlings; and **(C)** 6-week-old, senescent *Arabidopsis* plants, from WT and *erd7-1* and/or *erd7-2* mutant lines, as indicated by labels. Scale bar = 20 μm. Shown also in **(B,C)** in the graphs on the right are the quantifications of LD numbers per area and LD sizes. Values of LD numbers in **(B)** are averages ± SD from three biological replicates, with each replicate consisting of two leaf samples from 3–4 plants per line. Values of LD numbers in **(C)** are averages ± SD from two biological replicates of 10 plants per line. LD diameters were calculated using the same data set (i.e., micrographs) and are presented as the percentage of LDs in three size classes: <0.5 μm (small), 0.5–1.0 μm (intermediate), and >1.0 μm (large); refer also to key. Overall, there were no significant differences in either LD numbers per area or LD sizes in leaves of 15-day-old seedlings or 6-week-old (senescing) plants from WT and both the *erd7-1* and *erd7-2* mutant lines, based on Student’s *t*-tests (*p* ≤ 0.05). Similarly, based on images presented in **(A)** and others, no obvious qualitative differences in LD numbers or sizes were observed between WT and *erd7-1* mutant seeds and seedlings.

To begin to study any potential effects of *ERD7* disruption on LDs in Arabidopsis plants, we analyzed LDs with BODIPY staining and CLSM in dry seeds and seedlings during post-germinative growth, as well as in leaves of 15-day-old seedlings, all of which are organs that possess LDs with relatively high consistency in terms of their number and morphology and, thus, are often employed in studies of plant LDs (Gidda et al., 2016). Notably, RT-PCR-based analysis of *ERD7* gene expression in WT Arabidopsis confirmed that *ERD7* is expressed, albeit at varied levels, in a wide range of organs at different stages throughout plant growth and development, including in dry seeds, seedlings, mature plant leaves, and senescing leaves (Supplementary Figure 5A), and consistent with publicly-available Arabidopsis microarray expression data for *ERD7* (and other *SEN* genes; Supplementary Figure 5B). As shown in Figure 5A, CLSM analysis of BODIPY-stained cotyledons found no apparent visible differences in LD abundance and morphology in WT and *erd7-1* dry (mature) seeds or in seedlings 1, 2, or 4 days after the onset of germination, when storage oil (e.g., TAGs) and LDs are degraded to support seedling establishment. Similarly, visual comparisons and quantifications of BODIPY-stained LDs in 15-day-old seedlings from WT and both *erd7* mutant lines revealed no significant differences in LD numbers or size distributions (i.e., small, intermediate, and large-sized LDs; Figure 5B).

We also assessed the potential effects of *ERD7* disruption on LDs in senescing leaves of 6-week-old Arabidopsis plants, since ERD7 was previously reported to be enriched at LDs based on a proteomics analysis of Arabidopsis senescing leaves (Brocard et al., 2017). As shown in Figure 5C, visual comparisons and quantifications of BODIPY-stained LDs in senescent leaves from WT and both *erd7* mutant lines, similar to results from seedlings and seeds (Figures 5A,B), revealed no significant differences in LD numbers or size distributions in the *erd7* mutant lines compared with WT. However, as addressed in the Discussion section, whether the disruption in expression of *ERD7* and/or other *SEN* genes alters LD homeostasis in Arabidopsis during stress conditions, such as drought stress, is an important open question that requires further detailed investigation.

### Several Putative ERD7-Interacting Proteins Participate in Plant Stress Responses

Given that the results presented in Figure 5 indicate that ERD7 does not appear to play an essential role in LD homeostasis in plants, at least not in the organs and conditions examined, we sought to gain insight to possible function(s) of ERD7 by searching for potential interacting proteins, especially since many of the processes that LD-associated proteins are involved with are known to rely on protein-protein interaction networks (Kolkhof et al., 2017). Toward that end, a Y2H screen was conducted using ERD7 as bait along with a cDNA expression library derived from a variety of Arabidopsis tissues. In total, 36 candidate ERD7-interacting proteins were identified, as assessed by the relative growth of yeast (prey) transformants under selective conditions (Supplementary Table 3).

**Figure 6:**
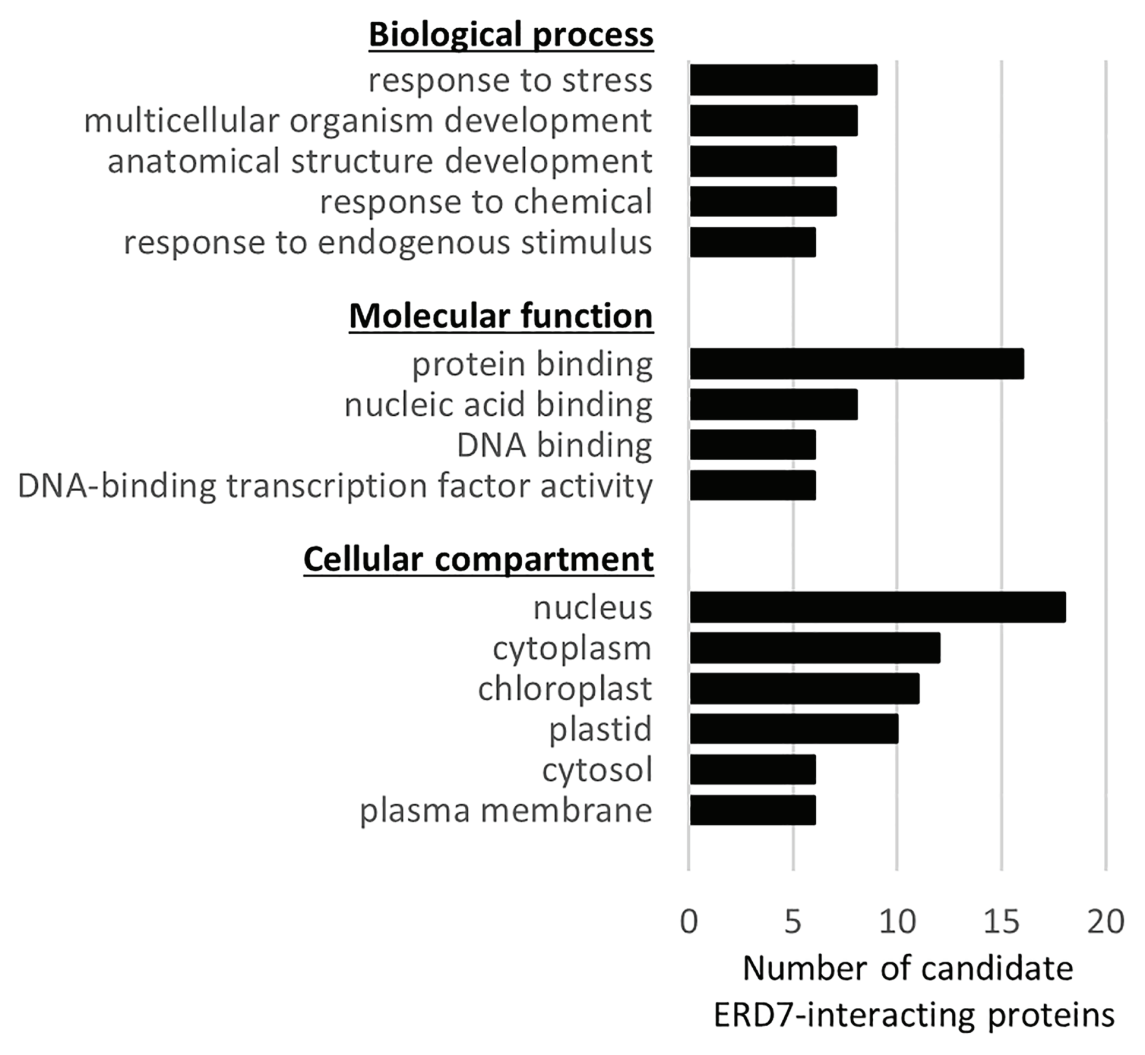
Functional categorization, based on Gene Ontology (GO) terms, of candidate ERD7-interacting proteins identified in a Y2H screen. Each of the 36 Arabidopsis proteins identified in the Y2H screen using ERD7 as bait (refer to Supplementary Table 3 for a list of all of the candidate ERD7-interacting proteins) were assigned GO terms based on the functional categorization of genes using the GO Slim annotation search tool at TAIR. Shown for each GO category (i.e., “Biological process,” “Molecular function,” and “Cellular component”), is the number of candidate ERD7-interacting proteins with the assigned GO annotation that appeared in at least six or more proteins.

While none of the 36 candidate ERD7-interacting proteins identified are known LD proteins [i.e., proteins experimentally confirmed *via* microscopy to be localized to LDs in plant cells based on Ischebeck et al. (2020)], several of them were identified in our drought-stressed leaf LD proteome and/or in other previously-published Arabidopsis LD proteomics studies (Supplementary Table 3). Furthermore, several of the candidate ERD7-interacting proteins shared the same GO terms (GO Slim, as assigned by the TAIR GO annotation search) within the GO categories of “biological process,” “molecular function,” and/or “cellular compartment” (Figure 6; Supplementary Table 3), which, by extension, might reflect the general functions of ERD7. For example, several of the candidate proteins are annotated in the GO Slim category of “biological process” to be involved with “response to stress” (Figure 6), which is notable given that ERD7 has the same assigned GO Slim term, based on its upregulation during plant stress responses (Winter et al., 2007; Cheng et al., 2013; Rasmussen et al., 2013). Among these proteins was BINDING PARTNER OF ACCELERATED CELL DEATH 11 (ACD11) 1 (BPA1), which we identified as a relatively strong candidate interactor with ERD7 (Supplementary Table 3) and was also exclusively detected, like ERD7, in LD-enriched fractions isolated from drought-stressed Arabidopsis leaves (refer to Supplementary Dataset 2), as well as in other previously-published Arabidopsis LD proteomics studies (Kretzschmar et al., 2018, 2020). BPA1 is known to bind and stabilize ACD11 in plant immune defense and hypersensitive response pathways, which are elicited by biotic stresses (Petersen et al., 2009; Li et al., 2019). Other interesting candidate ERD7-interacting proteins included the relatively large number that were annotated in the GO Slim categories of molecular function and cellular compartment to be involved in DNA-binding transcription factor activity and localized to the nucleus, respectively (Figure 6). These included the entire S4 family of MYB transcription factors, namely MYB3, MYB4, MYB7, and MYB32 (Dubos et al., 2010), which function to suppress gene expression during plant stress responses (Fornalé et al., 2014; Zhou et al., 2017; Agarwal et al., 2020). As discussed below, these observations, as well as those for other candidate ERD7-interacting proteins we identified, collectively raise intriguing questions about the potential role of ERD7 in the link between LDs and plant stress responses.

## Discussion

### ERD7 Is a Type II LD Protein That Targets to LDs *via* Its SD

The ongoing identification of novel LD-localized coat proteins affords us more tools to study LD-related processes in plants. Here, we show that a protein commonly associated with plant stress responses, ERD7 (Cheng et al., 2013; Rasmussen et al., 2013), is enriched with LDs isolated from drought-treated Arabidopsis leaves (Table 1), and targets to LDs when transiently- or stably-expressed in *N. benthamiana* and/or Arabidopsis (Figures 1, 2). While full-length ERD7 localized to both the cytosol and LDs, its C-terminal SD region alone localized almost entirely to LDs (Figure 2). This striking difference in subcellular localization indicates that the SD region of ERD7 is responsible for its LD localization, and further suggests that the N-terminal region potentiates LD localization of the full-length protein. Such regulated or potentiated protein localization might involve changes in protein conformation, due to the presence of post-translational modifications or perhaps through binding of a protein partner(s) that masks or reveals a protein targeting sequence (Bauer et al., 2015).

Lipid droplet coat proteins across eukaryotes are known to associate with LDs by two general mechanisms: (i) *via* a hydrophobic hairpin sequence that embeds into the neutral lipid core of the LD and is employed by so-called type I LD proteins or (ii) associating with the LD surface *via* protein-protein and/or protein-phospholipid head group interactions, which is used by type II LD proteins (Kory et al., 2016). ERD7 is assumed to be a type II LD protein because it lacks a predicted hydrophobic region that could form a hairpin. Type II LD proteins are known to associate with LDs *via* either an amphipathic α-helix, a lipid modification, and/or by binding to another LD coat protein(s) (Kory et al., 2016). Consistent with this, ERD7 contains at least two regions within its SD sequence that have propensity to form an amphipathic α-helix (amino acids 308–325 and 355–372), as determined by HeliQuest (Gautier et al., 2008), and the GPS-Lipid server (Xie et al., 2016) predicts a putative S-palmitoylation site at the cysteine residue at position 281 in ERD7, a post-translational modification known to be responsible for proper localization of other LD coat proteins (Kory et al., 2016). However, whether either or both of these putative signals and/or interactions with other LD coat proteins contributes to the LD localization of ERD7 in plant cell, remains to be determined.

### SENA and SENB Proteins Target to LDs or Mitochondria, Respectively

The localization of full-length ERD7 and the ERD7 SD region to cytosol and/or LDs (Figure 2) prompted us to investigate the subcellular localization of other SD-containing (i.e., SEN) proteins in plants. Land plants contain multiple SEN proteins separated into two distinct subfamilies, referred to in this study as SENA and SENB proteins (Figures 3A,B). Arabidopsis contains six SEN proteins, with three in each subfamily, including ERD7, which is a SENA-type protein (i.e., SENA1; Figures 3A,B). Unlike ERD7, which localized to both cytosol and LDs, transient expression of full-length Arabidopsis SENA2 and SENA3 in *N. benthamiana* leaves resulted in localization exclusively to the cytoplasm (Figure 4). Expression of the SENA2 and SENA3 SD regions alone; however, resulted in targeting almost exclusively to LDs (Figure 4), similar to what was observed for the ERD7 SD (Figure 2). The results together suggest that plant SENA-type proteins exhibit dual localization between cytosol and/or LDs, and that, as mentioned above, their subcellular distribution might be influenced by conformational changes that expose targeting signals within their SD regions. Expression of a full-length SENB-type protein (i.e., SENB2) in plant cells resulted also in localization to the cytosol, and surprisingly, the SD region of SENB2, as well as SENB1, did not target to LDs. Instead, both SENB1-SD and SENB2-SD targeted to mitochondria (Figure 4). Thus, the SEN protein family in plants appears to contain two distinct subfamilies whose members show potential for regulated distribution between cytosol and LDs or mitochondria, respectively. Notably, there are no obvious sequence motifs or conserved regions that are shared between the SDs in the LD-localized Arabidopsis SENA proteins and spartin, but absent in the SDs of the mitochondrial-localized SENB proteins. There are also no predicted mitochondrial targeting signals (based on the Plant-mPLoc and DeepLoc prediction programs) in the SDs of the Arabidopsis SENB proteins. Interestingly, ERD7 has been also reported to bind anionic phospholipids, such as phosphatidic acid (Barajas-Lopez et al., 2020), which is present in the LD monolayer (Thiam et al., 2013). It is plausible, therefore, that ERD7 (and the other Arabidopsis SENA proteins) localizes to LDs *via* binding anionic phospholipids on the LD surface, and similarly, mitochondrial targeting of SENB proteins could be mediated by binding to a different anionic phospholipid(s). How the SDs in these proteins are able to distinguish between different organelle locations in plant cells remains to be determined.

To determine whether this dual localization pattern might be an evolutionarily conserved feature of SEN-type proteins, we transiently expressed the human SEN protein spartin in plant cells. Notably, spartin is the only SD-containing protein found in humans (Figure 3A), where it localizes to LDs (Eastman et al., 2009; Urbancyzk and Enz, 2011), but also targets to mitochondria and other subcellular locations (Bakowska et al., 2007; Edwards et al., 2009; Joshi and Bakowska, 2011; Karlsson et al., 2014). When expressed in *N. benthamiana* leaf cells, the localization of spartin was similar to that of Arabidopsis SENA2 and SENA3, with full-length spartin localized to the cytosol, and the SD region alone targeting to LDs (Figure 4). This conservation of targeting suggests that the SD region of spartin might bind directly to LDs using biophysical features of LDs that are conserved between plants and animals, or by binding to an unidentified LD protein(s) conserved across plants and animals. Further, the differential localization of spartin to LDs or mitochondria in mammalian cells is interesting given that Arabidopsis SENA SDs localize to LDs and the SENB SDs localize to mitochondria. This suggests that plant SENA/SENB proteins might have diverged evolutionarily to have distinct subcellular localizations (i.e., LDs or mitochondria) and that multiple proteins evolved to carry out functions homologous to that of spartin in humans. However, it also cannot be ruled out that the SDs in plant SENA/SENB proteins might similarly localize to distinct subcellular organelles in different cellular and/or physiological contexts, since we only analyzed SD localization in one expression system, cell-type, and condition (i.e., transient-expression in *N. benthamiana* leaf epidermal cells subjected to standard growing conditions). Hence, the detailed characterization of the subcellular targeting signals in ERD7 and other plant SEN proteins and their potential modulation by cell type and/or stress conditions will be undoubtedly an important avenue of future investigation.

### ERD7 Is Stress-Inducible and May Serve as a Link Between LDs and Stress Responses

While it is well-known that *ERD7* is transcriptionally upregulated by a variety of abiotic stresses, including drought, salt, and cold (Winter et al., 2007; Cheng et al., 2013; Rasmussen et al., 2013; refer also to Arabidopsis microarray expression data for *ERD7* and other *SEN* genes in response to various stresses shown in Supplementary Figure 5C), studies on its possible molecular function(s) have only recently been carried out. That is, Barajas-Lopez et al. (2020) showed that ERD7 protein expression is similarly induced by cold and salt stress, and Arabidopsis mutants lacking *SENA2* and *SENA3* and heterozygous for *ERD7* (as the homozygous triple mutants were embryonic lethal) accumulated more anthocyanins during cold stress and leaves were more susceptible to freezing, perhaps due to decreased membrane flexibility. While the precise role(s) for ERD7 (and other plant SEN proteins) in membrane homeostasis during stress responses remains to be determined, our results from the Y2H screen conducted using ERD7 as bait (Figure 6; Supplementary Table 3) provide insight to help guide future studies. For instance, ERD7 potentially interacts with all four members of the S4 MYBs family, which are nuclear transcriptional repressors known to regulate phenylpropanoid biosynthesis (Zhou et al., 2017; Wang et al., 2020). It is possible, therefore, that ERD7 might regulate these transcriptional repressors by sequestering them in the cytosol, perhaps even at LDs, barring them from entering the nucleus. This premise is attractive given that other LD proteins have been shown to regulate nuclear protein factors by binding to and sequestering them on cytosolic LDs (Li et al., 2012; Ueno et al., 2013).

Among the other candidate ERD7-interacting proteins identified in our Y2H screen were two RING-type E3 ubiquitin ligases, SINAT2 and ATL72 (Supplementary Table 3). These proteins are notable given that human spartin has been shown to recruit E3 ubiquitin ligases to LDs and is proposed to function in controlling LD turnover by regulating the polyubiquitination and breakdown of LD proteins (Edwards et al., 2009; Hooper et al., 2010). This could point to an analogous role for ERD7 in controlling the turnover of LD proteins in plants, particularly during stress responses. In addition, the identification of BPA1 as a putative ERD7-interacting protein (Supplementary Table 3) is also notable, since BPA1 interacts with and stabilizes ACD11, which is a sphingolipid transfer protein involved in the plant immune defense and hypersensitive response pathways that are elicited by biotic stress (Petersen et al., 2009; Li et al., 2019). Since LDs are known to be involved in generation of lipid signals in eukaryotes, including in plants (Ischebeck et al., 2020), it is possible, therefore, that LD-localized ERD7 facilitates this process in response to stress. Whether ERD7 (or other Arabidopsis SEN proteins) serves to modulate MYB transcription factors, SINAT2/ATL72 E3 ubiquitin ligases, and/or BPA1/ACD11 activity remains to be determined. Of course, it will be important to first confirm in plant cells the putative interactions of ERD7 with these proteins or any of the other candidate ERD7-interacting proteins, we identified by Y2H analysis (Supplementary Table 3). Nonetheless, based on the results collectively presented in this study, ERD7 should serve as a unique tool for future studies aimed at elucidating the functional link between stress responses and LD biology in plants.

## Accession Numbers

Arabidopsis Genome Initiative (AGI) identifier numbers for the coding sequences of the Arabidopsis proteins examined in this study were obtained from TAIR and are as follows: *ERD7* (AT2G17840.1), *SENA2* (AT4G35985.1), *SENA3* (AT3G51250.1), *SENB1* (AT3G21600.1), *SENB2* (AT4G15450.1), *SENB3* (AT3G21590.1), *LPEAT1* (AT1G80950), and *TUBULIN 4* (AT5G44340). The National Center for Biotechnology Information (NCBI) Consensus CDS identifier numbers for *H. sapiens SPARTIN* and TBSV *P19* are NM_015087.5 and CAC01278.1, respectively.

## Data Availability Statement

The datasets presented in this study can be found in online repositories. The names of the repository/repositories and accession number(s) can be found in the article/Supplementary Material.

## Author Contributions

ND, DS, MM, SS, SG, and TI performed the experiments. KS and GB performed the mass spectrometry. ND and RM wrote the manuscript with input from JD, KC, and TI. All authors contributed to the article and approved the submitted version.

### Conflict of Interest

The authors declare that the research was conducted in the absence of any commercial or financial relationships that could be construed as a potential conflict of interest.

## Footnotes

^1^http://dnasu.org

^2^http://genome.arizona.edu/crispr2/

^3^http://ppdb.tc.cornell.edu/dbsearch/subproteome.aspx

^4^http://string-db.org

^5^http://arabidopsis.org

^6^http://uniprot.org

^7^http://phytozome.jgi.doe.gov

^8^https://www.ebi.ac.uk/interpro/entry/InterPro/IPR009686/

^9^http://bar.utoronto.ca

^10^https://www.ebi.ac.uk/Tools/msa/clustalo/

^11^http://heliquest.ipmc.cnrs.fr

^12^http://cbs.dtu.dk/services/DeepLoc-1.0/

^13^http://www.csbio.sjtu.edu.cn/bioinf/plant-multi/

^14^http://lipid.biocuckoo.org
