# Supplementary material for: Arabidopsis thaliana EARLY RESPONSIVE TO DEHYDRATION 7 Localizes to Lipid Droplets via Its Senescence Domain
Source: Front Plant Sci. 2021 Apr 14;12:658961. doi: 10.3389/fpls.2021.658961 (PMC8079945; doi:10.3389/fpls.2021.658961)
Supplement: Supplementary file 3 [file Image_1.pdf]

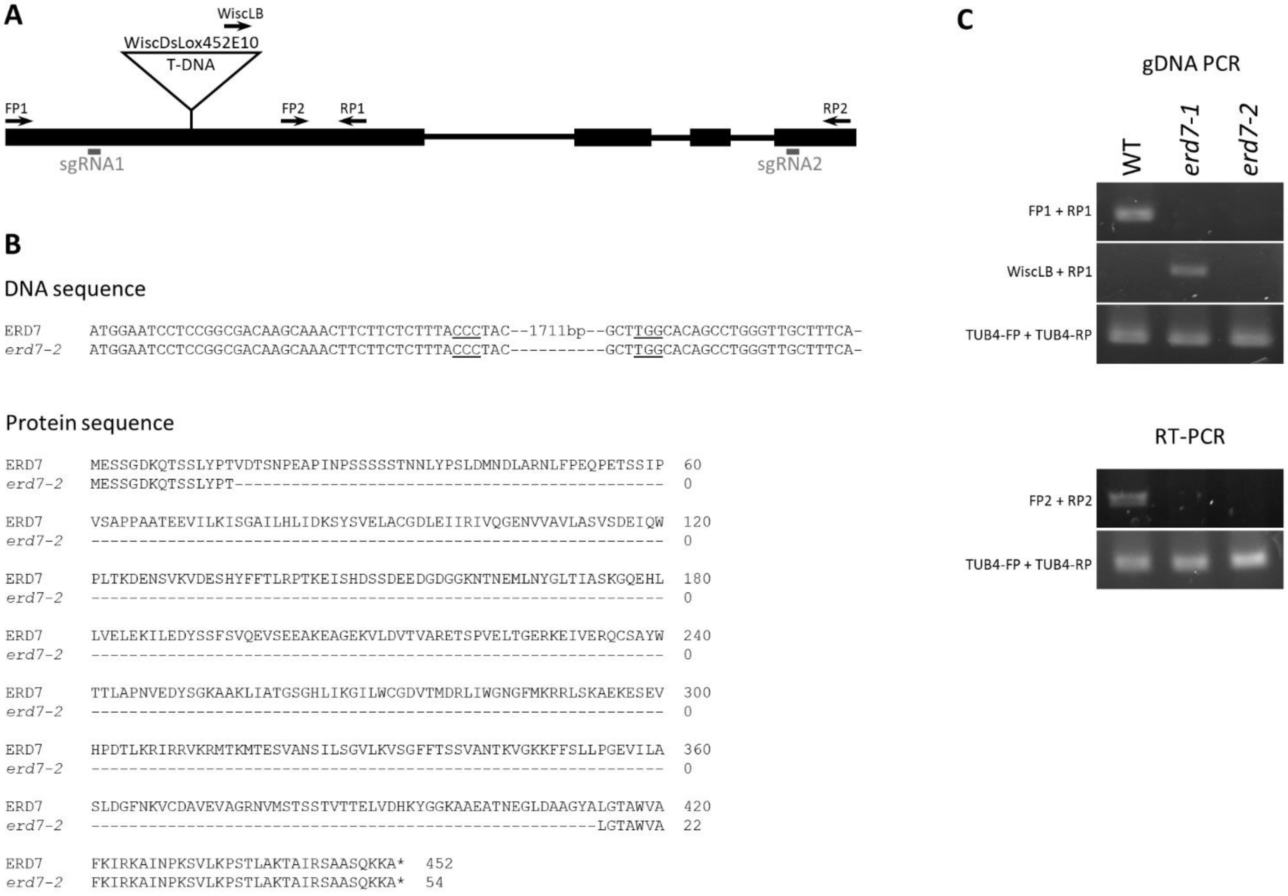

Supplementary Figure 1 | Generation and characterization of *ERD7* transgenic Arabidopsis mutant lines. (A) Illustration depicting the Arabidopsis *ERD7* ORF (AT2G17840.1), with the 5' end on the left, based on information provided at TAIR. Indicated are the *ERD7* gene exons (boxes) and introns (lines) and the relative positions of the T-DNA insert and sgRNA regions targeted for CRISPR/Cas9 genome editing in the corresponding single-copy, T_3 homozygous *erd7-1* and *erd7-2* mutant lines. Also shown are the relative positions of the primer pairs used for genotyping and RT-PCR analysis in (C). **(B)** Comparison of the nucleotide and deduced polypeptide sequences of the *ERD7* gene and protein in WT and the *erd7-2* mutant line, indicating the expected 1711 nucleotide deletion in the *ERD7* gene (and in the corresponding transcript) and resulting 398 amino acid deletion in the encoded protein in the *erd7-2* mutant line. The CRISPR/Cas9 protospacer adjacent motif is underlined in the *ERD7* WT and *erd7-2* mutant DNA sequences. Deduced amino acid sequences for the WT and mutant ERD7 proteins were aligned using the ClustalO algorithm (ebi.ac.uk/Tools/msa/clustalo) (Madeira et al., 2019). **(C)** PCR and RT-PCR analysis of *erd7-1* and *erd7-2* mutant lines. Shown is the PCR analysis of gDNA (upper panel) and RT-PCR analysis of mRNA (lower panel) extracted from rosette leaves of 15-day-old WT, *erd7-1,* and *erd7-2* plants and assessed with the indicated primer pairs; refer to (A) for the positions of the primer pairs. Refer also to **Supplementary Table 1** for all primer sequences used in generation and characterization of the two *erd7* mutant lines. PCR products and RT-PCR products were analyzed by DNA gel electrophoresis and ethidium bromide staining. Note the absence of PCR and RT-PCR products corresponding to the *ERD7* gene and *ERD7* expression (i.e., transcripts), respectively, in both mutant lines, as well as the presence of the T-DNA in the *erd7-1* mutant, as expected. Arabidopsis *TUB4* was used an endogenous control.

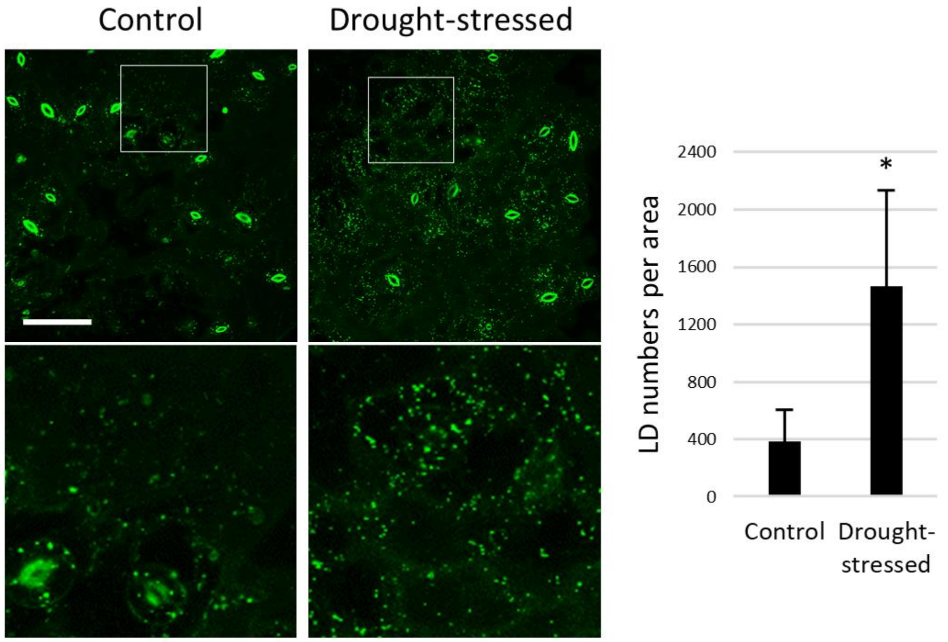

Supplementary Figure 2 | Proliferation of LDs in drought-stressed Arabidopsis leaves. Shown are representative CLSM images (2D projections of z-stacks) of BODIPY-stained LDs in leaves of (WT) Arabidopsis plants were grown on soil for four weeks before regular watering was either continued (i.e., control plants) or withheld (i.e., drought-stressed), as indicated by labels. Boxes in the top row represent the portion of the leaf shown at higher magnification in the bottom row, showing the relative increase in LDs in drought-stressed leaves. Scale bar = 50 µm. Shown also in the graph on the right are the quantifications of LD numbers per area in leaves from control and drought-stressed plants. Values of LD numbers are averages ± standard deviation from 10 leaf samples (i.e., two leaf areas from 5 different plants) per treatment. Asterisk indicates a statistically significant difference ($P \leq 0.001$), as determined by a Student's *t*-test.

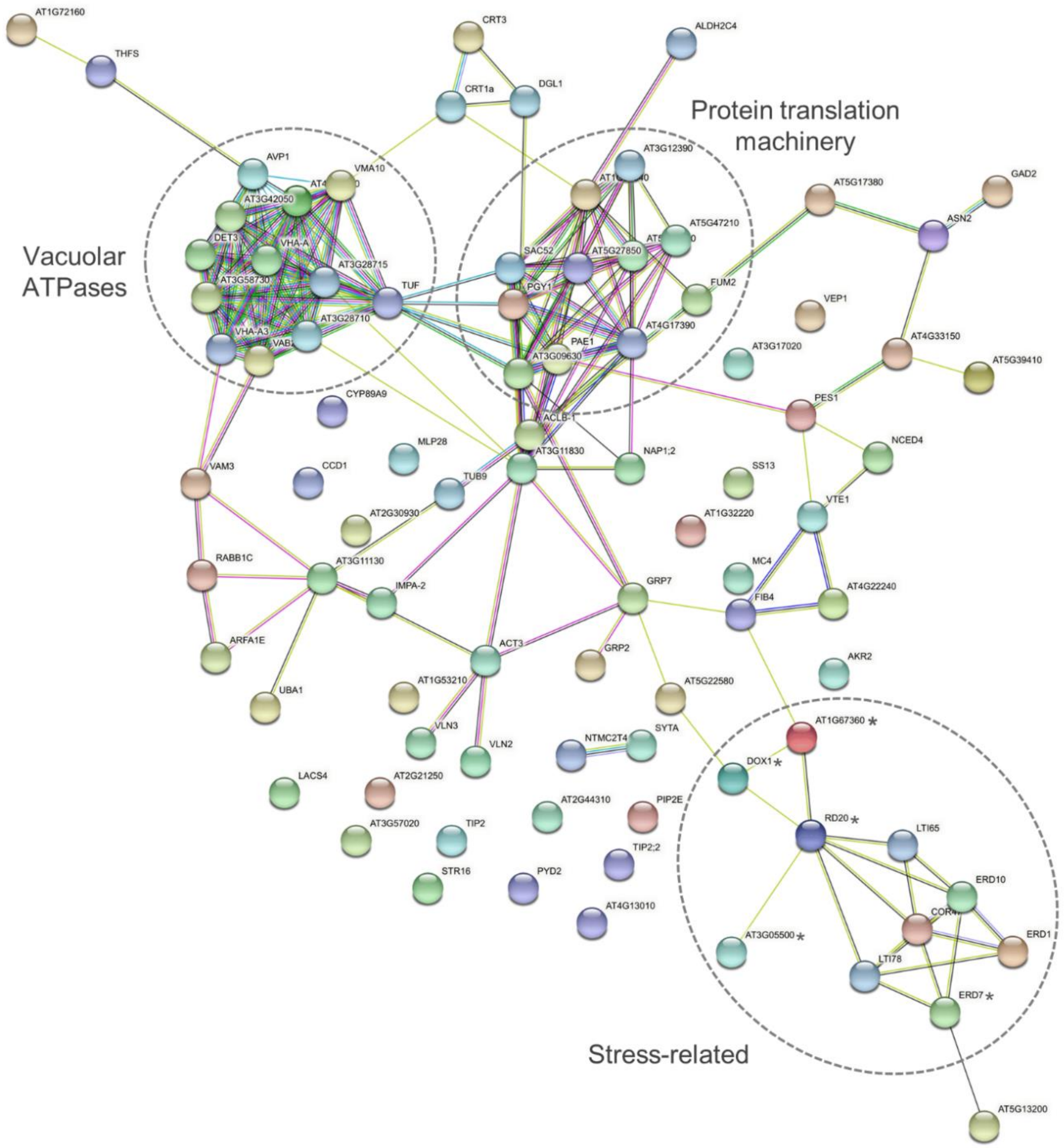

Supplementary Figure 3 | STRING functional protein association network of selected proteins enriched with LDs isolated from drought-stressed Arabidopsis leaves. All of the 89 candidate LD-enriched proteins listed in **Supplementary Dataset 4** were analyzed using the STRING informatics tool (string-db.org) (Szklarczyk et al., 2019), as described in the Bioinformatics section in the Materials and Methods. Each circle represents an individual protein in the cohort and the color of the lines connecting proteins (circles) indicate the various types of interaction evidence, based on annotations provided at the STRING database: cyan, interaction known from curated database; magenta, interaction experimentally determined; green, interaction predicted by gene neighborhood; red, interaction predicted by gene fusions; blue, interaction predicted by gene co-occurrence; yellow, relationship predicted by text-mining; black, relationship predicted by co-expression; and lavender, relationship predicted by protein homology. Also indicated are three conspicuous clusters of proteins (refer to stippled circles) enriched with proteins annotated (based on STRING and TAIR) as vacuolar ATPase subunits or involved in either protein translation (e.g., ribosomal subunits and eukaryotic initiation factors) or plant stress response. Note that several of the proteins in the ‘stress-related’ protein cluster include known LD proteins (e.g., RD20, LDAP1/3 and DOX1), as well as ERD7.

A

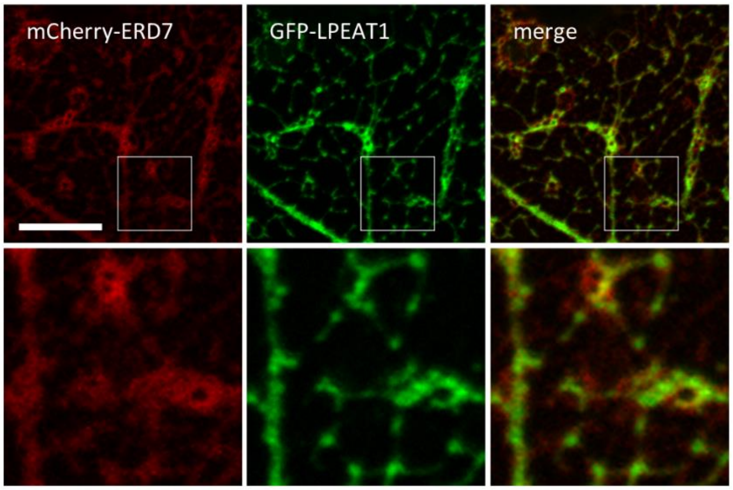

B

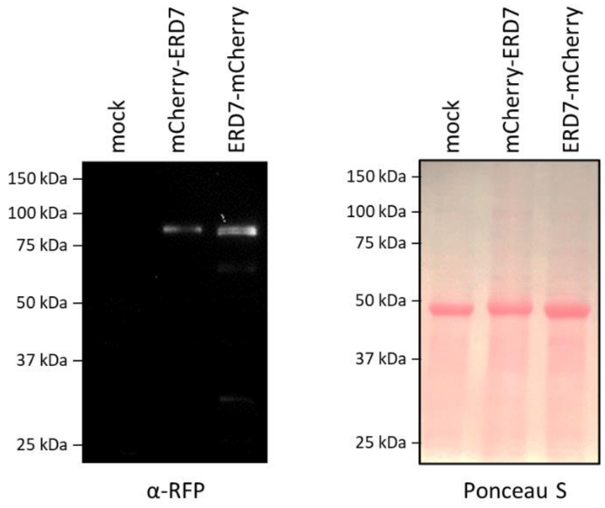

Supplementary Figure 4 | Subcellular localization and immunoblotting of ectopically-expressed ERD7 in *N. benthamiana* leaves. (A) Representative CLSM images (z-sections) of *Agrobacterium*-infiltrated *N. benthamiana* leaf epidermal cells co-transformed with mCherry-ERD7 and the ER marker protein GFP-LPEAT1 (as indicated by the panel labels). Shown also is the corresponding merged image. Boxes in the top row represent the portion of the cell shown at higher magnification in the bottom row. Note the diffuse fluorescence attributable to mCherry-ERD7 in the cytosol is mostly distinct from the reticular fluorescence attributable to ER-localized GFP-LPEAT1. Scale bar = 10 µm. **(B)** Immunoblot analysis of soluble (total) protein extracts from *N. benthamiana* leaves transiently-(co)transformed (via *Agrobacterium* infiltration) with either p19 alone ('mock') or p19 and mCherry-ERD7 or ERD7-mCherry, as indicated by labels. Shown on the left is the immunoblot incubated with anti-RFP antibodies and, on the right, is the corresponding Ponceau S staining of the same blot prior to immunodetection, illustrating that there was relatively equal protein loading per sample (see Immunoblotting section of the Materials and Methods for additional details on immunoblotting procedures). Positions of molecular mass markers are also indicated. Note that the most predominant immunoreactive bands observed in the mCherry-ERD7 and ERD7-mCherry samples (but not in the 'mock sample) are those consistent with the expected size for both full-length fusion proteins (i.e., ~ 77 kDa).

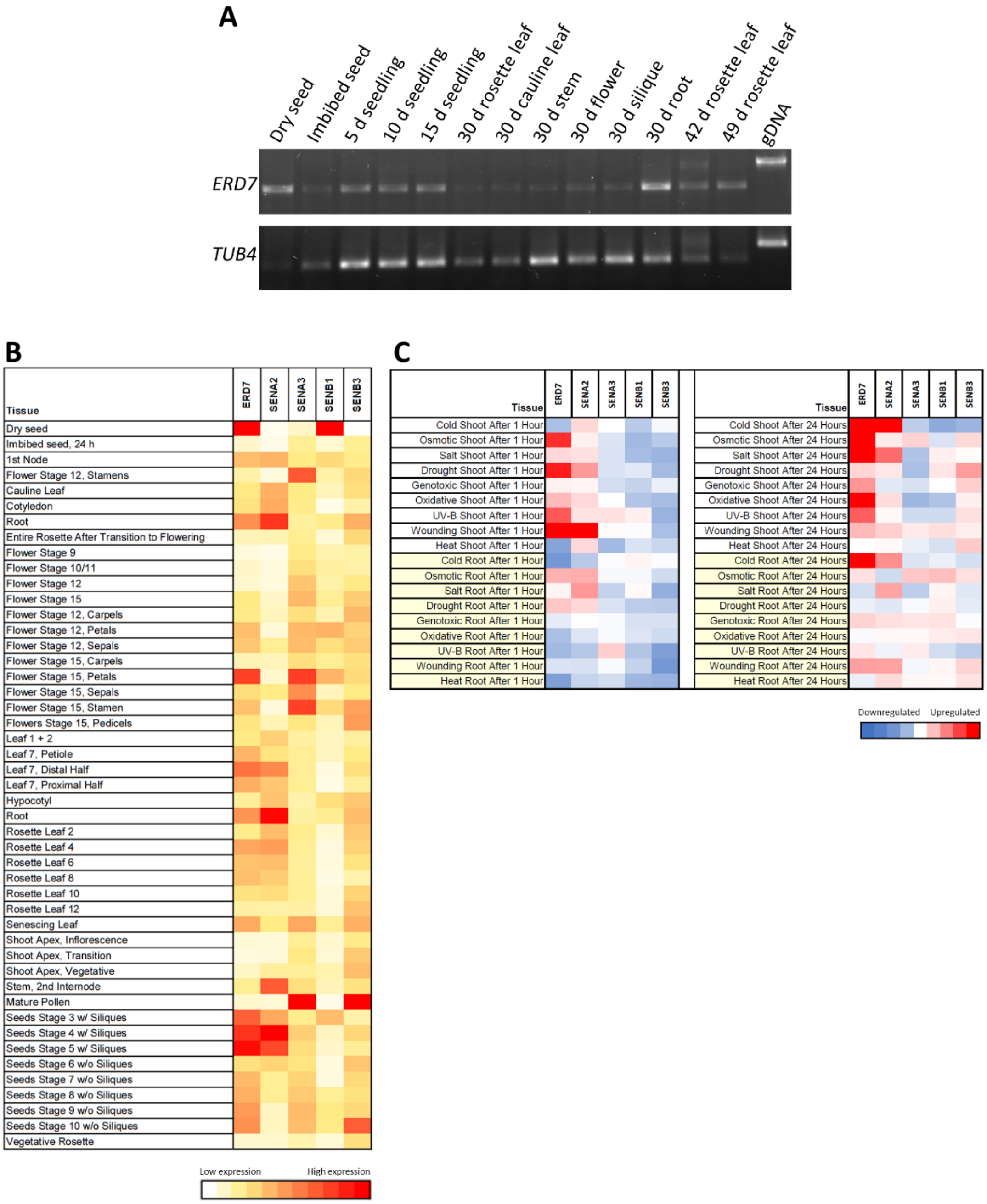

Supplementary Figure 5 | Expression of *ERD7* and other *SEN* genes in Arabidopsis. (A) RT-PCR analysis of *ERD7* gene expression in various organs and developmental stages in Arabidopsis (d, day; gDNA, genomic DNA). *TUBULIN 4* (TUB4) was used as an endogenous control. **(B) and (C)** Microarray-based expression profile of *ERD7* and other selected *SEN* genes in Arabidopsis in (B) various organs and tissues and developmental stages and (C) seedlings subjected to 1 h or 24 h of an abiotic stress treatment, as indicated by labels. Heat maps were generated from publicly-available microarray datasets available at the Arabidopsis eFP Browser hosted at BAR (Winter et al., 2007) and details on growth conditions and stress treatments are described in Kilian et al. (2007). Keys at the bottom of each heat map indicate the correlation between color and scaled log-fold changes in gene expression, including in (C) where changes in gene expression (i.e., downregulation or upregulation) due to stress treatments are those compared to an untreated control. Seedling data in (C) is presented for both shoots (upper rows) and roots (lower rows). Note that microarray data for *SENB2* is not available since this gene is not present on the ATH1 whole genome chip.
